# HIF-1α Stabilization Increases miR-210 Eliciting First Trimester Extravillous Trophoblast Mitochondrial Dysfunction

**DOI:** 10.3389/fphys.2019.00699

**Published:** 2019-06-06

**Authors:** Lauren Anton, Ann DeVine, Erzsebet Polyak, Anthony Olarerin-George, Amy G. Brown, Marni J. Falk, Michal A. Elovitz

**Affiliations:** ^1^Maternal and Child Health Research Center, Department of Obstetrics and Gynecology, Perelman School of Medicine at the University of Pennsylvania, Philadelphia, PA, United States; ^2^Division of Human Genetics, Department of Pediatrics, The Children’s Hospital of Philadelphia, Philadelphia, PA, United States; ^3^Department of Pharmacology, Institute for Translational Medicine and Therapeutics, Perelman School of Medicine at the University of Pennsylvania, Philadelphia, PA, United States

**Keywords:** preeclampsia, extravillous trophoblast, miRNA, miR-210, mitochondrial respiration, ISCU, NDUFA4, SDHD

## Abstract

Preeclampsia is associated with first trimester placental dysfunction. miR-210, a small non-coding RNA, is increased in the preeclamptic placenta. The effects of elevated miR-210 on placental function remain unclear. The objectives of this study were to identify targets of miR-210 in first trimester primary extravillous trophoblasts (EVTs) and to investigate functional pathways altered by elevated placental miR-210 during early pregnancy. EVTs isolated from first trimester placentas were exposed to cobalt chloride (CoCl_2_), a HIF-1α stabilizer and hypoxia mimetic, and miR-210 expression by qPCR, HIF1α protein levels by western blot and cell invasion were assessed. A custom TruSeq RNA array, including all known/predicted miR-210 targets, was run using miR-210 and miR-negative control transfected EVTs. Mitochondrial function was assessed by high resolution respirometry in transfected EVTs. EVTs exposed to CoCl_2_ showed a dose and time-dependent increase in miR-210 and HIF1α and reductions in cell invasion. The TruSeq array identified 49 altered genes in miR-210 transfected EVTs with 27 genes repressed and 22 enhanced. Three of the top six significantly repressed genes, NDUFA4, SDHD, and ISCU, are associated with mitochondrial function. miR-210 transfected EVTs had decreased maximal, complex II and complex I+II mitochondrial respiration. This study suggests that miR-210 alters first trimester trophoblast function. miR-210 overexpression alters EVT mitochondrial function in early pregnancy. Mitochondrial dysfunction may lead to increased reactive oxygen species, trophoblast cell damage and likely contributes to the pathogenesis of preeclampsia.

## Introduction

Preeclampsia (PE), a hypertensive disorder of pregnancy, affects 5–8% of pregnancies making it one of the world’s leading causes of maternal and fetal morbidity and mortality United States Department of Health and Human Services. (1991). PE is a disorder characterized clinically by the presence of maternal hypertension (greater than 140/90), proteinuria and edema after 20 weeks gestation. The maternal PE phenotype can vary widely from mild, the presence of only slight increases in blood pressure, to severe, the presence of significant end-organ damage (in the liver and kidneys). The spectrum of symptoms associated with PE has resulted in the theory that there are two forms of PE, early- (before 34 weeks gestation) and late-onset PE with early-onset PE correlating to more severe clinical manifestations of the disorder (Redman, 2017). Early-onset/severe PE is associated with higher rates of intrauterine growth restriction, preterm birth, and perinatal death indicating that placental dysfunction could be a major contributor to the development of PE. While the exact pathophysiology of PE remains unclear, it is believed that PE stems from “placental inefficiency” a term used to describe shallow trophoblast invasion of the maternal uterine spiral arteries during the first trimester of pregnancy which results in decreased placental perfusion and consequently, placental hypoxia/ischemia. The hypoxic placenta stimulates the release of factors resulting in systemic maternal endothelial dysfunction and the clinical syndrome of PE (Maynard et al., 2003; Levine et al., 2004, 2006).

Normal placental function and development is dependent on oxygen tension and requires the availability of differing amounts of oxygen (depending on the trimester) for cell growth, proliferation, invasion and metabolism (Genbacev et al., 1996, 1997). The first trimester placenta functions under low oxygen conditions until vascularization is established (Caniggia et al., 2000). However, in preeclampsia, it is believed that there is a period of prolonged hypoxia due to reduced trophoblast invasion resulting in increased HIF-1α stabilization (Caniggia et al., 2000). HIF1α, a master regulator of the cellular hypoxic response, has been shown to control mitochondrial function and is essential for the repression of mitochondrial respiration during hypoxia (Agani et al., 2000; Angela et al., 2008). Mitochondria, often known as the powerhouse of the cell, are responsible for consuming oxygen and releasing the energy needed for normal cellular function. Placental mitochondrial dysfunction during gestation can often lead to excessive reactive oxygen species (ROS) formation leading to increased oxidative stress which has been shown to contribute to the abnormal placental function observed in the pathogenesis of PE (Wang and Walsh, 1998, 2001; Many et al., 2000). Specifically, the upregulation of ROS has been shown to inhibit prolyl hydroxylases and stabilize hypoxia inducible factor (HIF) proteins (Niecknig et al., 2012) which have varied biological effects on placental function including alterations in the glycolytic pathway (Semenza et al., 1994), angiogenesis (Kelly et al., 2003), and invasion (Highet et al., 2015) among many others. While there is an established connection between mitochondrial dysfunction, placental dysfunction, and PE (Holland et al., 2017), the exact mechanisms contributing to the disruption in mitochondrial activity are unclear. This is especially critical during the first trimester of pregnancy, at a time where a reduction in mitochondrial activity and, hence, placental function could ultimately lead to pregnancy disorders such as PE (Watson et al., 1998).

Many of the cellular processes in placental development, including trophoblast differentiation, migration, invasion, proliferation, apoptosis, vasculogenesis/angiogenesis, and cellular metabolism, have been shown to be regulated by microRNAs (miRNAs) (Hayder et al., 2018). miRNAs are highly conserved, small (22 nucleotide), non-coding RNA molecules known to cause translational gene repression (Bartel, 2004). Over the last decade, many miRNAs have been identified in the human placenta and have been shown to be increased in PE placentas (Pineles et al., 2007; Zhu et al., 2009). One of the most consistently identified PE-associated miRNAs is miR-210, a well-described “hypoxamir” (Chan and Loscalzo, 2010; Zhang et al., 2012). miR-210 is induced in response to hypoxia in a HIF-1α dependent manner. We, and others, have previously shown that miR-210 is increased in the placenta and serum of women with PE and that elevated miR-210 inhibits primary first trimester extravillous trophoblast (EVT) invasion, a process highly dependent on the availability of oxygen, energy and metabolic activity (Zhang et al., 2012; Anton et al., 2013). Additional studies have shown a mechanistic link between miR-210, mitochondrial associated genes and mitochondrial respiration (Chan et al., 2009; Chen et al., 2010; Puisségur et al., 2010). A comprehensive study performed by Muralimanoharan et al. (2012) provided evidence linking elevated miR-210 to decreased mitochondrial respiration in third trimester trophoblasts and villous tissue. However, as PE is thought to be initiated with placental dysfunction in the first trimester, the role of miR-210 in placental and mitochondrial function during this early time point is of critical importance and remains unknown.

While several studies have shown that miR-210 is increased in PE placentas and has effects on placental function, comprehensive investigation of the genes altered by miR-210 in the first trimester placenta is limited. Therefore, the objectives of this study were to identify downstream targets of miR-210 in first trimester primary EVT cells and to investigate the functional biological pathways altered by elevated miR-210 in the placenta during early pregnancy. We hypothesize that overexpression of placental miR-210 alters genes regulating first trimester EVT function resulting in defective placentation contributing to PE. In order to investigate this, we (1) utilized a custom TruSeq RNA array to identify genes directly and significantly altered by miR-210 overexpression in first trimester EVT cells and (2) assessed the function of these altered genes by showing that elevated miR-210 significantly reduces mitochondrial respiration in first trimester EVT cells.

## Materials and Methods

### Ethics Statement

This study was carried out in accordance with the recommendations of the Institutional Review Board at the University of Pennsylvania (IRB# 700943, protocol # 817472). This study does not meet the criteria for the definition of a human subject meaning a living individual about whom an investigator conducting research obtains (1) data through intervention or interaction with the individual, or (2) identifiable private information. Therefore this study was approved for exemption from IRB oversight as no contact was made with the patient and no identifying patient information was recorded for any of the first trimester placentas collected.

### Cell Culture

Primary EVT cells were isolated from first trimester villous tissue using a protocol that has been established and well documented by Graham et al. (Graham et al., 1992; Getsios et al., 1998; Anton et al., 2012). Briefly, finely minced chorionic villi collected from ten de-identified elective first trimester pregnancy termination placentas (less than 12 weeks) were cultured at 37°C in RPMI 1640 medium containing 20% charcoal-stripped (steroid-free) fetal bovine serum (FBS). EVT cells, which outgrow from attached villous fragments, were separated from villous tissue during washing and passaging of the cells. The isolated EVT cells were maintained in RPMI 1640 medium containing 20% FBS and 1% penicillin (100 U/mL)/streptomycin (100 U/mL) solution. The EVT cells used in our experiments were characterized by immunostaining for trophoblast cell markers, cytokeratin-7, 8 and 18, HLA-G and integrin alpha-1 (Neudeck et al., 1997; Koi et al., 2002; Anton et al., 2012; Gomez et al., 2012). These results are similar to those obtained by other investigators using the same EVT isolation methods and confirm the purity of the EVT cell preparations (Graham et al., 1992; Getsios et al., 1998).

### EVT Cobalt Chloride Treatment

Extravillous trophoblasts were plated at 2.5 × 10^5^ cells/well in 24-well plates. Forty eight hours later, the cells were treated with 0–400 μM of CoCl_2_ (Sigma, St. Louis, MO, United States) a HIF-1α stabilizer and hypoxia mimetic (Piret et al., 2002; Yaung et al., 2008), or vehicle control (water) dissolved in RPMI 1640 for 6 h (*n* = 4). Additionally, a time-dependent response to CoCl_2_ (100 μM) was performed from 0 to 24 h (*n* = 4). After treatment, the media was aspirated, the cells were washed with PBS and 500 μL of Trizol (Invitrogen, Life Technologies, Grand Island, NY, United States) was added to each well for future miRNA extraction.

### Western Blots

Extravillous trophoblasts were plated at 1 × 10^5^ cells/well in a 6-well plate in 2 mL of media (1640 RPMI + 20% FBS). Forty eight hours later, the cells were treated with 100 μM of a CoCl_2_ solution in RPMI for the indicated times. The cells were rinsed with ice cold PBS at the time of harvesting and whole cell protein lysates were extracted with ice-cold RIPA buffer. Protein concentrations were estimated with the DC protein assay (Bio-Rad, Hercules, CA, United States) according to the manufacturer’s protocol. Twenty micrograms of each protein lysate was resolved via SDS-PAGE and transferred to PVDF membranes. The membranes were blocked for 1 h at room temperature with blocking buffer (5% milk, 0.5% Tween 20, 1× Tris-buffered saline) and then probed with HIF-1α (Cell Signaling, Beverly, MA, United States; 3716S) or alpha tubulin (Abcam, Cambridge, MA, United States; ab7291) primary antibodies in blocking buffer at 4°C overnight. Membranes were rinsed twice each with wash buffer (0.5% Tween 20, 1× Tris-buffered saline) then blocking buffer, then probed with anti-rabbit or anti-mouse IgG HPR-linked secondary antibodies (GE Healthcare, Piscataway, NJ, United States) in blocking buffer for 30 min at room temperature. Membranes were rinsed 4 times in wash buffer for 10–15 min each. Chemiluminescence ECL reagent (Perkin Elmer, Waltham, MA, United States) was added to the membranes according to the manufacturer’s instructions. Membranes were then exposed to X-ray film.

### Matrigel Invasion Assay

The invasiveness of primary EVT cells through an extracellular matrix was measured using a commercially available cell invasion assay kit (Chemicon, Temecula, CA, United States). Briefly, EVT cells were treated with CoCl_2_ (100 μM) for 24 h prior to being used for the invasion assay and for an additional 72 h in the media in the top chamber during the invasion assay. After 72 h, the non-invading cells and the ECMatrix gel from the upper surface of the inserts was removed using a cotton-tipped swab. Invasive cells on the lower surface of the membrane were stained with 0.2% crystal violet for 20 min. The membranes were mounted onto microscope slides. Stained cells from five random microscope fields at 20× magnification were photographed, counted and analyzed. Data from experiments measuring EVT invasion are expressed as a percent of control.

### EVT Cell Transfection

Extravillous trophoblast cells isolated from 10 different de-identified placentas were individually plated at 2 × 10^5^ cells/well in 6-well plates in antibiotic-free 1640 RPMI media containing 20% FBS. The next day, the cells were transfected with miRNA mimics (40 nM). Hsa-miR-210 (miR-210) and miR-negative control (miR-neg, non-targeting control) miRNA mimics were purchased from Applied Biosystems (Life Technologies). Lipofectamine RNAiMAX (Invitrogen) was used for the transfection of the miRNA mimics according to the manufacturers’ protocol. Cells were transfected for 24 h and maintained under normal growth conditions. Transfection efficiency was verified by qPCR (Supplementary Figure S1).

### mRNA Isolation From EVT Cells

miR-210 and miR-neg transfected EVT cells were collected in Trizol and underwent phenol-chloroform extraction. The resulting aqueous phase was further column purified with the miRNeasy kit (Qiagen) according to the manufacturer’s protocol for total RNA isolation including small RNAs. RNA concentration was determined via a NanoDrop 2000 Spectrophotometer (NanoDrop^TM^, Rockland, DE, United States). The quality of the RNA was assessed by the RNA Integrity Number (RIN) using the Agilent BioAnalyzer 2100 (Agilent Technologies, Santa Clara, CA, United States) and miR-210 transfection efficiency of each EVT cell line was confirmed prior to moving forward with additional experiments (Supplementary Figure S2). RNA isolated from these cells was then used on the TruSeq Targeted RNA Array and for qPCR validation of the array.

### Creating the Custom TruSeq Targeted RNA Expression Array

A custom TruSeq Targeted RNA Expression array (Illumina, San Diego, CA, United States) was created to include all known or predicted human downstream targets of miR-210 as identified by the TargetScan Database^1^ (version 6.2). After referencing the TargetScan Database, 586 possible miR-210 target genes were identified. Using Illumina DesignStudio, an interface for custom probe design, 576 of these genes had available oligos that were added to the array. In addition to the miR-210 target genes, five endogenous controls (YWHAZ, UBC, RPL30, GUSB, and RPL37A) were included on the array. These endogenous controls were chosen after running a TaqMan Array Endogenous Controls Plate (Thermo Fisher Scientific, Applied Biosystems, Foster City, CA, United States) which contains 32 possible endogenous control genes. Five of these 32 genes were chosen based on the overall level of expression and that the expression level remained unchanged after miR-210 transfection. There were a total of 581 target genes on the final array (Supplementary Table S1).

### Running the TruSeq Targeted RNA Expression Array

Fifty nanograms of total RNA from each EVT line (*n* = 10 per transfection group representing 10 independent placentas) was converted to cDNA using Protoscript II (New England Biolabs), DTT (10 mM final concentration) and buffer supplied in the TruSeq Targeted RNA Custom Panel Kit (Illumina). Reactions were incubated in a thermal cycler using the following profile: 25°C for 5 min; 42°C for 15 min; 95°C for 10 min; hold at 4°C. The cDNA was hybridized to the custom panel of primers designed to the target areas using reagents from the kit and the following profile: 70°C for 5 min; 68°C for 1 min; 65°C for 2.5 min; 60°C for 2.5 min; 55°C for 4 min; 50°C for 4 min; 45°C for 4 min; 40°C for 4 min; 35°C for 4 min; 30°C for 4 min; hold at 30°C. Following washing at room temperature with agitation to remove unbound oligos, the annealed primers were extended and ligated to adjacent products by incubation at 37°C for 45 min. The extension/ligation products were amplified using primers that add index sequences for sample multiplexing as well as common adapters required for cluster generation. Reactions were amplified with the following profile: 95°C for 2 min; 25 cycles of 98°C for 30 s, 62°C for 30 s, 72°C for 60 s; 72°C for 5 min; hold at 10°C. Amplification reactions were purified with AMPure XP beads, washed twice with 80% ethanol, dried and then eluted with resuspension buffer. Supernatant was collected, equal volumes mixed to create a pool of 40 samples and the mixture run on an Agilent DNA-1000 Bioanalyzer chip for quantitation. Based on the area of the library peak from 100 to 300 bp, the pool was normalized to 4 nM. The library was denatured and clustered according to standard MiSeq guidelines at a target concentration of 12 pM and a single 50-bp read with dual indexing (6- and 8-bp reads) was conducted using version 3 chemistry. Primary alignment and analysis of the reads was performed using the MiSeq Reporter Targeted RNA workflow on BaseSpace^2^.

### Analysis of the TruSeq Targeted RNA Expression Array

Raw counts per target for 20 samples were normalized and analyzed for differential expression using DESeq2 running in R (Love et al., 2014). Contrasts were calculated for miR-210 vs. miR-neg. An adjusted *p*-value was calculated by DESeq2 for FDR (False Detection Rate) by the method of Benjamini and Hochberg. Log2 transformed normalized expression intensities were imported into Partek Genomics Suite (Partek, Inc., St. Louis, MO, United States) for visualizations including heat-maps and principal components analysis (PCA). Genes were considered differentially expressed between miR-neg and miR-210 if their adjusted *p*-value was lower than 0.05. Genes meeting the adjusted *p*-value cut off criteria were submitted to the Database for Annotation, Visualization, and Integrated Discovery (DAVID) Bioinformatics Resource version 6.8^3^ (Huang et al., 2008, 2009) to identify the functional biological pathways associated with those genes showing the largest decrease in expression after miR-210 transfection. Genes identified by DAVID as being associated with significant gene functional classifications via the functional annotation tool and/or the Gene Ontology (GO Terms) database were used to validate the TruSeq Targeted RNA Expression array and for downstream functional assays.

### cDNA Generation and qPCR for miRNA or mRNA Quantification

cDNA was generated from 1 μg of isolated RNA from transfect EVT cells (*n* = 10) using the miScript Reverse Transcription II kit (Qiagen) or high capacity cDNA reverse transcription kit (Thermo Fisher Scientific, Applied Biosystems) for SYBR Green (for miRNA measurement) or TaqMan (for mRNA measurement) primers, respectively. qPCR was performed on the 7900HT Real-Time PCR System (Applied Biosystems) using the miScript SYBR Green PCR kit (Qiagen) or TaqMan Universal PCR Master Mix (Applied Biosystems) according to the manufacturers’ protocols. The ΔΔCT (SYBR Green PCR) or standard curve (TaqMan PCR) method was used for relative expression quantification using the RQ manager software v2.4 (Applied Biosystems). For SYBR Green PCR, the endogenous reference gene RNU6B was used for miRNA quantification from EVT cells. All primer sets were purchased from Qiagen: miR-210 (MS00003801) and RNU6B (MS00014000). For TaqMan PCR, the relative abundance of the target of interest was divided by the relative abundance of 18S in each sample to generate a standardized abundance for the target transcript of interest. All primer sets were purchased from Applied Biosystems: NDUFA4, SDHD, ISCU, and 18S.

### High Resolution Respirometry in Permeabilized EVTs

Extravillous trophoblast cells (*n* = 7, representing seven independent placentas) were grown in their normal culture media and transfected with miR-negative control and miR-210 as described in the methods above. The cells were then prepared for respiratory capacity analysis by high resolution polarography using an Oxygraph-2K (Oroboros, Austria), as previously described (Kuznetsov et al., 2008; Pesta and Gnaiger, 2012; Zhang et al., 2013). After trypsinization, the cells were washed with Dulbecco’s phosphate buffered saline following one wash with respiration medium MIR05 and then re-suspended in MIR05 medium containing 0.5 mM EGTA, 3 mM MgCl_2_ × 6H_2_O, 20 mM taurine, 10 mM KH_2_PO_4_, 20 mM HEPES, 1 g/L BSA, 60 mM potassium-lactobionate, 110 mM sucrose, pH = 7.1. Control (miR-neg transfected) and miR-210 transfected EVTs were simultaneously analyzed in two separate chambers in a 2 ml volume containing 1 million cells in each chamber. Experiments were performed as previously described (Zhang et al., 2013) with slight modifications. Cells were permeabilized according to the published protocol using digitonin as the permeabilizing agent (Kuznetsov et al., 2008) after optimization of the digitonin amount as described (Pesta and Gnaiger, 2012). Respiration was measured at 37°C in respiration medium (MIR05), where substrates and inhibitors (purchased from Sigma) were added in the following order with the final concentration denoted in parentheses: malate (5 mM), glutamate (10 mM), ADP (2.5 mM), cytochrome C (10 μM), succinate (10 mM) stepwise titration of carbonyl cyanide-p-trifluoromethoxyphenylhydrazone (FCCP) (Sigma) uncoupler in 0.25 μM increments as needed, Rotenone (0.5 μM), Antimycin A (5 μM). Data was analyzed using DatLab4 and DatLab6 (Oroboros, Austria) software.

### Statistical Analysis

For all experiments, excluding results from the TruSeq Targeted RNA Expression array which were analyzed using methods described above, statistical analyses were performed with GraphPad Prism Software (version 7.0, San Diego, CA, United States). For data that were normally distributed, *t*-tests or one way analysis of variance (ANOVA) were used. If the data were not normally distributed then the non-parametric Mann–Whitney test was used. Results with *p* < 0.05 were considered statistically significant.

## Results

### Hypoxia Mimetic Cobalt Chloride Induces miR-210 Expression and Inhibits EVT Invasion

**FIGURE 1:**
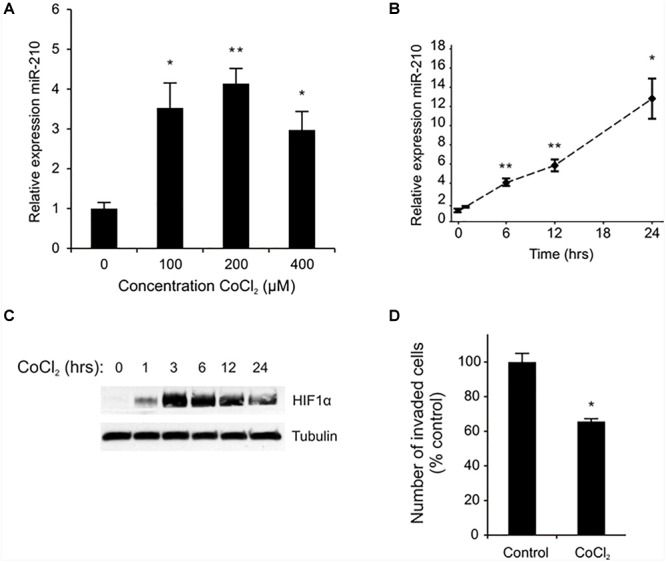
CoCl_2_ regulates EVT miR-210 expression and invasion. miR-210 expression was measured in EVT cells treated with the indicated doses of CoCl_2_ by qPCR **(A)**. Time course of miR-210 mRNA **(B)** and HIF-1α protein **(C)** expression in EVTs treated with CoCl_2_ (100 μM) was performed between 0 and 24 h of exposure. An invasion assay was performed on EVTs treated with 100 μM CoCl_2_ **(D)**. Values are mean ± SEM. *n* = 4 for **(A,B)**, *n* = 3 for **(D)**, ^∗^*p* ≤ 0.05, ^∗∗^*p* < 0.01 compared to non-treated controls.

To determine if miR-210 expression is HIF-1α/hypoxia regulated in EVTs, we treated cells with the HIF-1α stabilizer and hypoxia mimetic, cobalt chloride (CoCl_2_) (28–30). CoCl_2_ induced miR-210 expression in a dose (Figure 1A) and time (Figure 1B) dependent manner. miR-210 expression was significantly induced in EVTs treated with 100, 200, and 400 μM of CoCl_2_ for 6 h (*p* < 0.05; Figure 1A). Using the lowest effective dose of CoCl_2_ (100 μM), we also performed a time course experiment and found a steady increase in miR-210 expression up to 24 h after treatment (Figure 1B). Next, to confirm CoCl_2_ was indeed mimicking hypoxia in the EVTs, we measured HIF-1α protein expression in cells exposed to CoCl_2_ over the course of 24 h (Figure 1C). Expression of HIF-1α protein was evident as early as 1 h after treatment, peaked at 3 h, and remained high as long as 24 h post treatment. Next, we assessed if CoCl_2_, like miR-210, altered trophoblast invasion. Indeed, treatment of EVTs with CoCl_2_ decreased EVT invasion by 40% relative to the negative control (*p* = 0.014; Figure 1D).

### TruSeq Targeted RNA Expression Array Analysis

**FIGURE 2:**
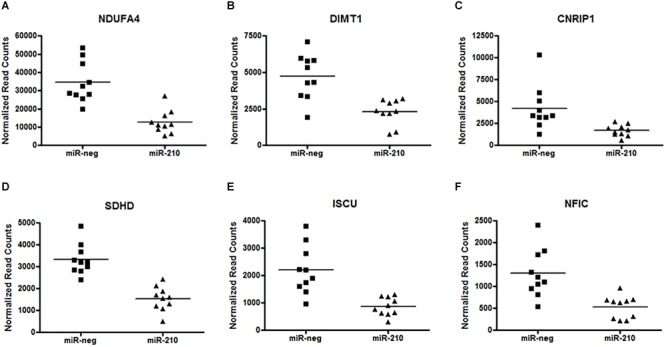
Repression of miR-210 direct target genes. Analysis of the custom TruSeq Targeted RNA array revealed 49 altered (27 repressed and 22 enhanced) genes. The top six significantly repressed genes after EVT transfection of miR-210 are NDUFA4 **(A)** (adj. *p* = 1.40E-24), DIMT1 **(B)** (adj. *p* = 9.26E-18), CNRIP1 **(C)** (adj. *p* = 9.26E-18), SDHD **(D)** (adj. *p* = 1.79E-16), ISCU **(E)** (adj. *p* = 2.12E-09), and NFIC **(F)** (adj. *p* = 1.04E-07). Raw counts per target for 20 samples (*n* = 10 per group) were normalized and analyzed for differential expression (versus miR-negative transfected controls) using DESeq2. The adjusted *p*-value was calculated by DESeq2 for FDR (False Detection Rate) by the method of Benjamini and Hochberg.

Utilizing a custom TruSeq Targeted RNA Expression array, we investigated altered gene expression in primary first trimester EVT cells transfected with either miR-negative control (miR-neg) or miR-210. The result of this array showed that 494 of the 581 genes present on the array had sequencing reads with a range of 1–2.75 million reads per gene. Using the results from the TruSeq Targeted RNA Expression array, we compared the mean average of sequencing-depth-normalized counts and calculated the log2 fold change (as provided by the DESeq2 analysis) between miR-neg and miR-210 transfected EVTs (Supplementary Table S1). Of the 494 genes with sequencing reads, including only those genes with adjusted *p*-values below 0.05, we identified 49 genes with altered expression after miR-210 transfection. Making up the 49 altered genes were 27 repressed genes and 22 enhanced genes (Table 1). From this list of 49 genes, we chose to focus on the six genes with the most significant decreases in expression after miR-210 transfection including NDUFA4 (adj. *p* = 1.40E-24, Figure 2A), DIMT1 (adj. *p* = 9.26E-18, Figure 2B), CNRIP1 (adj. *p* = 9.26E-18, Figure 2C), SDHD (adj. *p* = 1.79E-16, Figure 2D), ISCU (adj. *p* = 2.12E-09, Figure 2E), and NFIC (adj. *p* = 1.04E-07, Figure 2F). Functional annotation and gene ontology analysis of these genes, using DAVID Bioinformatics Resources, showed that three of these six genes, NDUFA4, SDHD, and ISCU, were associated with the mitochondrion and metabolic processes. Both NDUFA4 and SDHD cluster together in respiratory electron transport chain, cellular respiration and oxidative phosphorylation functional groups. Given the analogous biological function of these three genes (Table 2), they were used to validate expression changes found by the TruSeq Targeted RNA Expression array by an independent method (qPCR) and to further investigate if decreased expression of these genes resulted in a functional change.

**Table 1:** Genes with significantly altered expression after extravillous trophoblast transfection with miR-210.

Gene name	Accession number	Log2 fold change	Adjusted *p*-value
NDUFA4	NM_002489	–1.25	1.40E-24
DIMT1	NM_014473	–0.88	9.26E-18
CNRIP1	NM_015463	–1.01	9.26E-18
SDHD	NM_003002	–0.96	1.79E-16
ISCU	NM_014301	–1.05	2.12E-09
NFIC	NM_205843	–1.03	1.04E-07
GDE1	NM_016641	–1.03	2.72E-07
SRP19	NM_003135	0.64	5.21E-07
ATXN10	NM_013236	0.34	1.48E-05
SUPT7L	NM_014860	0.67	1.14E-04
TTC13	NM_024525	–0.87	1.36E-04
IGF2BP2	NM_006548	0.44	1.44E-04
STMN1	NM_203401	0.36	2.83E-04
SLC7A11	NM_014331	–0.51	3.42E-04
FLNC	NM_001458	–0.86	3.42E-04
SDF2	NR_045585	–0.65	5.32E-04
CD59	NM_203330	0.28	5.61E-04
PXDC1	NM_183373	–0.63	5.89E-04
RIT1	NM_006912	0.49	7.28E-04
TNPO1	NM_153188	–0.47	7.59E-04
RPL22	NM_000983	0.28	1.01E-03
ANKRD13A	NM_033121	–0.45	1.26E-03
CELF1	NM_001172639	–0.56	1.38E-03
CENPN	NM_001100625	0.53	1.39E-03
RAB7A	NM_004637	0.28	2.09E-03
KAT6A	NM_001099413	–0.68	3.17E-03
B4GALT5	NM_004776	–0.87	3.69E-03
NAT14	NM_020378	0.50	5.02E-03
SCARA3	NM_182826	–0.67	7.27E-03
FCHSD2	NM_014824	–0.73	7.43E-03
RAB3B	NM_002867	–0.57	0.010
CNP	NM_033133	0.61	0.012
SF3B3	NM_012426	0.48	0.012
FUNDC2	NM_023934	0.46	0.013
LDOC1L	NM_032287	0.40	0.015
AGPAT2	NM_006412	–0.86	0.015
KIAA0930	NM_015264	–0.66	0.024
SPRED2	NM_181784	–0.58	0.024
FGFRL1	NM_001004358	–0.84	0.025
ZBTB34	NM_001099270	0.65	0.032
ZNF148	NM_021964	0.46	0.034
WDFY2	NM_052950	0.45	0.034
MRPL36	NM_032479	–0.71	0.038
PTPN21	NM_007039	–0.77	0.038
PGAM5	NM_138575	–0.33	0.038
PDAP1	NM_014891	0.37	0.038
LYN	NM_002350	0.82	0.040
KCMF1	NM_020122	0.45	0.042
SHB	NM_003028	0.54	0.044

**Table 2:** Mitochondrial genes repressed by miR-210.

Gene	Gene name	Gene function
NDUFA4	NADH-ubiquinone oxidoreductase MLRQ subunit	NADH dehydrogenase activity and oxidoreductase activity transfers electrons from NADH to the respiratory chain.
SDHD	Succinate dehydrogenase complex	Membrane-anchoring subunit of succinate dehydrogenase (SDH) that is involved in complex II of the mitochondrial electron transport chain. Responsible for transferring electrons from succinate to ubiquinone (coenzyme Q).
ISCU	Iron-sulfur cluster assembly enzyme	Involved in the assembly or repair of the iron sulfur clusters that are incorporated into enzymes involved in energy production, including mitochondrial respiratory complexes I, II, and III.

### qPCR Validation of the TruSeq Targeted RNA Expression Array

**FIGURE 3:**
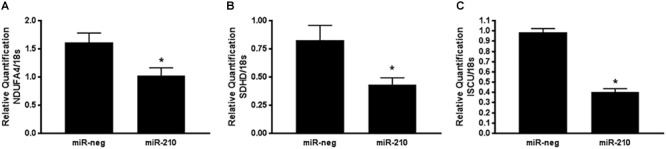
Positive qPCR validation of a subset of genes identified by the TruSeq Targeted RNA array. Three genes identified by the TruSeq Targeted RNA array were chosen for validation by qPCR based on their mitochondria-associated functional annotation. NDUFA4 **(A)**, SDHD **(B)**, and ISCU **(C)** mRNA expression was reduced after miR-210 transfection of EVTs. Values are mean ± SEM. *n* = 10 representing EVT cells from 10 independent placentas. ^∗^*p* < 0.05 compared to miR-negative (miR-neg) transfected control.

In order to validate the TruSeq Targeted RNA Expression array, qPCR was run using isolated RNA from the same 10 miR-neg/miR-210 transfected EVT lines as were run on the array. qPCR was run on the three genes identified above as being significantly repressed by miR-210 and involved in mitochondrial respiration, NDUFA4, SDHD and ISCU. NDUFA1 (Figure 3A, *p* = 0.0208), SDHD (Figure 3B, *p* = 0.0165), and ISCU (Figure 3C, *p* < 0.0001) were significantly decreased after miR-210 transfection confirming the array results.

### Increased miR-210 Reduces Mitochondrial Respiration

**FIGURE 4:**
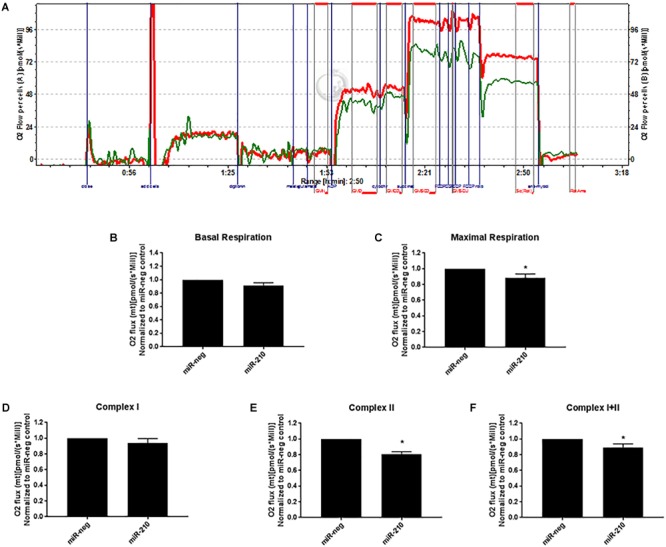
Elevated miR-210 decreases EVT mitochondrial respiration. Using high resolution respirometry (Oroboros Instruments), EVTs transfected with miR-210 (green line) compared to miR-negative controls (miR-neg, red line) showed significant reductions in mitochondrial respiration as evidenced by the representative experimental tracing of oxygen consumption **(A)**. Elevated miR-210 expression significantly reduced maximal respiration **(C)** and respiration from complex II **(E)** and complex I+II **(F)**. No changes were seen in basal respiration **(B)** or complex I **(D)**. Levels of O2 flux were normalized to miR-negative controls. Values are mean ± SEM. *n* = 7 representing EVT cells from seven independent placentas. ^∗^*p* < 0.05 compared to miR-negative (miR-neg) transfected control.

Using an Oroboros Oxygraph-2K, high resolution mitochondrial respirometry was determined in EVTs transfected with miR-negative control or miR-210. A representative respiration tracing shows a decrease in the rate of respiration in miR-210 (green line) versus miR-neg (red line) transfected cells (Figure 4A). EVTs transfected to overexpress miR-210 showed no change in basal respiration (Figure 4B) but had reduced maximal (Figure 4C, *p* = 0.0377) respiration rates than cells transfected with miR-negative control. Since NDUFA4, SDHD, and ISCU predominately function in complex I and II of the mitochondria, we focused on those two complexes of mitochondrial respiration. While complex I (Figure 4D) respiration was unchanged, complex II (Figure 4E, *p* < 0.0001) and complex I+II (Figure 4F, *p* = 0.0355) respiration were significantly reduced after transfection of miR-210.

## Discussion

This study provides evidence that (1) elevated miR-210 expression has the ability to alter EVT gene transcription in the first trimester of pregnancy, a time that is critical to normal placentation and fetal growth and (2) miR-210 plays a critical role in EVT mitochondrial function in the first trimester of pregnancy suggesting that alterations in miR-210 expression contribute to adverse obstetrical outcomes, especially PE. In this study, we have shown that overexpression of miR-210 in first trimester EVTs has the ability to repress or activate many genes across a wide range of biological functions with the most significant gene alterations being associated with mitochondrial function. Additionally, we show that increased miR-210 in the EVT cells decreases mitochondrial respiratory function during the first trimester of pregnancy. Therefore, the results of this study support our hypothesis that altered miR-210 expression results in EVT dysfunction that may contribute to the pathogenesis of PE.

Many studies have shown that miR-210 is elevated in the PE placenta suggesting a functional role for miR-210 in placental pathogenesis (Pineles et al., 2007; Zhu et al., 2009). Interestingly, miR-210 has been shown to be expressed predominately in the villous and EVT cells of the placenta with higher expression in the latter (Lee et al., 2011). Furthermore, miR-210 is widely known to be induced by hypoxia (Chan and Loscalzo, 2010), as is seen in PE placentas (Zhang et al., 2012), via the stabilization of HIF-1α. While this link between miR-210 and hypoxia has been definitively established in many tissues, the regulation of miR-210 by hypoxia in first trimester EVT cells has not been shown previously. The upregulation of miR-210 and HIF-1α after exposure to cobalt chloride, a HIF-1α stabilizer and hypoxia mimetic, in both a dose and time-dependent manner suggests that miR-210 is regulated similarly in first trimester EVT cells as has been shown in other human tissues. Additionally, the reduction in EVT cell invasion in the presence of HIF-1α stabilization suggests that miR-210 could be mechanistically contributing to decreased first trimester placental function. Notably, in a previous publication investigating the effects of miR-210 on placental function, we showed that elevated miR-210 significantly decreases first trimester EVT invasion (Anton et al., 2013). Collectively, these data provide evidence that the HIF-1α-mediated increase in miR-210 expression has the ability to significantly alter EVT cell function early in pregnancy creating a biologically plausible mechanism for the role of miR-210 in the pathogenesis of PE.

In an effort to determine the functional pathways altered by miR-210, many studies have investigated the effects of this miRNA on downstream target genes in various disease states. Previous studies have mostly chosen to focus on one or two genes known/predicted to be altered by miR-210, while other studies have worked to create miRNA-mRNA regulatory networks through the utilization of bioinformatics databases such as Gene Ontology, Ingenuity, and TargetScan (Ahn et al., 2017; Biró et al., 2017; Pan et al., 2018). In this study, we focused on identifying miR-210 target genes in primary first trimester EVT cells, the cell type most predominantly associated with the pathogenesis of PE due to defective invasion. In order to be as comprehensive and accurate as possible, we developed a custom TruSeq Targeted RNA array which included a complete list of known/predicted miR-210 gene targets. Utilizing this novel approach, this study was able to identify specific target genes that were most significantly altered in the presence of elevated miR-210. Only one other study has attempted to identify miR-210 target genes in first trimester placental trophoblast cells using a transformed commercially available cell line, Swan 71 (Straszewski-Chavez et al., 2009). Using an Affymetrix Human GeneChip, this study identified 408 differentially expressed genes in miR-210 transfected Swan 71 cells (Ahn et al., 2017). While the results of this study are useful when determining overall functional pathways that may be affected by miR-210, it remains unknown if these genes are direct targets of miR-210 or are altered by an indirect response to miR-210. Although a full list of differentially expressed genes was not provided, interestingly, the identification of genes altered by miR-210 does seems to be different between the two studies, as only two of the genes discussed in the manuscript, were similarly identified (NFIC and FCHSD2). This would suggest that a transformed trophoblast cell line reacts differently to epigenetic modifications by miR-210. The extent of the biological differences between first trimester primary EVT cells and the transformed Swan 71 cells is not completely known, however, several studies have found both similarities, including cytokine profile (Straszewski-Chavez et al., 2009), and differences, such as HLA expression (Apps et al., 2009) and DNA methylation (Novakovic et al., 2011) between the two cell types. These results provide evidence that differences in gene expression due to epigenetic modifications between primary and transformed trophoblast cells is biologically plausible.

Notably, the results of the custom TruSeq array identified genes with both increased (22 genes) and decreased (27 genes) expression after miR-210 transfection suggesting that not all of the gene targets predicted by TargetScan are direct targets of miR-210. In our study, three (NDUFA4, SDHD, and ISCU) of the top five most significantly repressed targets of miR-210 were genes involved in mitochondrial or metabolic function. In light of these results and in conjunction with previously published studies showing alterations in mitochondrial function in preeclamptic placentas (Wang and Walsh, 1998; Muralimanoharan et al., 2012; Vishnyakova et al., 2016), we chose to focus on investigating the role of miR-210 in mitochondrial respiration.

Mitochondria act as the primary energy producers of the cell and are regulators of cellular metabolism. These organelles use oxygen to produce ATP through a series of steps in the electron transport chain as well as oxidative phosphorylation which occurs through the respiratory complexes (I–V) found in the inner mitochondrial membrane. Genes such as NDUFA4, SDHD, and ISCU (that were found to be dysregulated by miR-210 in this study) have been shown to regulate the function of diverse mitochondrial processes. NDUFA4 (NADH dehydrogenase subcomplex) is historically associated with complex I of oxidative phosphorylation (Mishmar et al., 2006; Hayashi et al., 2009). SDHD (succinate dehydrogenase D) is one of the two transmembrane subunits of the succinate dehydrogenase complex II (Burger et al., 1996). ISCU (iron sulfur cluster assembly enzyme) is a scaffold protein for the *de novo* synthesis of iron-sulfur clusters within the mitochondria that are critical for electron transport and oxidation-reduction reactions (Tong and Rouault, 2000). Interestingly, similar to the results of this study, previous studies performed outside of the pregnancy field have shown that these three genes are directly regulated by miR-210. In a comprehensive study investigating the effects of miR-210 in lung cancer, the authors show that miR-210 directly targets NDUFA4 and SDHD (by 3′UTR assays) resulting in decreased complex II activity, enlarged mitochondria with a modified organization of cristae and altered mitochondrial membrane potential (Puisségur et al., 2010). miR-210 has also been shown to target ISCU in pulmonary arterial endothelial cells where low levels of ISCU (mediated by miR-210) resulted in decreased aconitase (TCA) and complex I activity with reduced mitochondrial respiration (Chan et al., 2009; Chen et al., 2010). While neither NDUFA4 nor SDHD have been associated with preeclampsia or placental dysfunction, a decrease in ISCU mRNA has been shown in the preeclamptic placenta (Muralimanoharan et al., 2012) and miR-210 has been shown to directly reduce ISCU expression in both Swan 71 and BeWo trophoblast cells lines resulting in siderosomal/intracellular iron accumulation and a reduction in Swan 71 cell invasion (Lee et al., 2011). In agreement with our results, these findings suggest that miR-210 directly targets NDUFA4, SDHD, and ISCU resulting in reduced expression of these genes and the consequent reduction in mitochondrial function. The reduced mitochondrial function observed in the presence of decreased NDUFA4, SDHD, and ISUC expression could be due to decreased electron transport and oxygen usage leading to reductions in mitochondrial respiration and increased ROS production which are placental pathologies previously shown to be found in PE.

In order to more clearly understand the effects of miR-210 on mitochondrial function and given that NDUFA4, SDHD, and ISCU alterations have significant effects on complex I and II of the electron transport chain, we chose to assess mitochondrial respiration as an indicator of the ability of first trimester EVTs to produce and utilize energy. Cellular respiration occurs through oxidative phosphorylation within the mitochondria where oxygen is transformed to ATP through a series of protein complexes known as the electron transport chain. Defects in the electron transport chain can produce ROS (superoxide and hydrogen peroxide) and free oxygen radicals (oxidative stress) which are well known to cause cellular damage and contribute to a large variety of human diseases including cardiovascular disease and cancer among many others. In PE, prolonged placental hypoxia due to defective trophoblast invasion in the first trimester has been associated with increased ROS production which is believed to contribute to the development of the maternal symptoms of this disease (Kimura et al., 2013; Schoots et al., 2018). In this study, elevated miR-210 resulted in decreased maximal, complex II and complex I+II mitochondrial respiration suggesting that miR-210 (likely through reductions in NDUFA4, SDHD, and ISCU) has the ability to cause defects in oxidative phosphorylation within first trimester EVT cells. Notably, a study done by Muralimanoharan et al. (2012) showed, similarly, that miR-210 transfected third trimester villous cytotrophoblasts had reduced mitochondrial oxygen consumption including a reduction in both basal and maximal respiration. The fact that both studies independently identified significant miR-210-mediated changes in mitochondrial genes in placental trophoblasts provides strong evidence that miR-210 contributes to trophoblast dysfunction throughout gestation. While the results of this prior study show that miR-210 transfected third trimester villous cytotrophoblasts exhibit defects in mitochondrial respiration, it remained unknown if the development of PE was mechanistically associated with mitochondrial dysfunction as a cause or effect of late gestation placental injury. The results of these two studies performed at two different gestational time points (first trimester vs. third trimester) would suggest that defects in mitochondrial function first develop early in pregnancy, as shown in this study, are propagated throughout gestation persisting at delivery. This would suggest that defects in mitochondrial function initiated in the first trimester of pregnancy are sufficient to result in significant early or late gestation placental dysfunction and, consequently, PE.

Overall this study provides evidence of a role for miR-210 in regulating first trimester EVT function by directly targeting genes responsible for mitochondrial respiration. Importantly, these results provide novel insight into the role of epigenetic modifications on placental function at a time in pregnancy that is most critical for normal placental development. As severe/early-onset PE is often thought to occur due to defects in placentation during the first trimester, it is biologically plausible to hypothesize that a reduction in EVT invasion leading to prolonged placental hypoxia (upregulation of HIF-1α) causes elevated miR-210 which directly targets NDFUA4, SDHD, and ISCU resulting in decreased mitochondrial respiration and, consequently, a decrease in ATP production and an increase in placental ROS. Future studies investigating this mechanism would be warranted in order to determine if therapeutic options focused on miR-210 or its downstream mitochondrial targets would prove useful in reducing the placental dysfunction associated with PE.

## Data Availability

The raw data supporting the conclusions of this manuscript will be made available by the authors, without undue reservation, to any qualified researcher.

## Author Contributions

LA wrote the manuscript and created the figures. LA, AB, and ME conceived and designed the experiments and contributed to the analysis and interpretation of all data. LA, AD, EP, and AO-G performed the experiments. EP and MF contributed to performing and analyzing the mitochondrial respiration experiments. All authors contributed to manuscript revision and approved the final manuscript.

## Conflict of Interest Statement

The authors declare that the research was conducted in the absence of any commercial or financial relationships that could be construed as a potential conflict of interest.

## Footnotes

1 http://www.targetscan.org

2 https://basespace.illumina.com

3 http://david.abcc.ncifcrf.gov
