# Supplementary material for: HIF-1α Stabilization Increases miR-210 Eliciting First Trimester Extravillous Trophoblast Mitochondrial Dysfunction
Source: Front Physiol. 2019 Jun 6;10:699. doi: 10.3389/fphys.2019.00699 (PMC6590495; doi:10.3389/fphys.2019.00699)
Supplement: Supplementary file 1 [file Data_Sheet_1.docx]

**HIF-1α stabilization increases miR-210 eliciting first trimester extravillous trophoblast mitochondrial dysfunction**

**Authors:** Lauren Anton^1*^, Ann DeVine^1^, Erzsebet Polyak^2^, Anthony Olarerin-George^3^, Amy G. Brown^1^, Marni J. Falk^2^, Michal A. Elovitz^1^

^1^ Maternal and Child Health Research Center, Department of Obstetrics and Gynecology, Perelman School of Medicine at the University of Pennsylvania, Philadelphia, PA 19104, USA.

^2^ Division of Human Genetics, Department of Pediatrics, The Children's Hospital of Philadelphia and University of Pennsylvania School of Medicine, Philadelphia, PA 19104, US

^3^ Department of Pharmacology and the Institute for Translational Medicine and Therapeutics, Perelman School of Medicine at the University of Pennsylvania, Philadelphia, PA 19104, USA.

**Supplementary Figure S1: miR-210 transfection time course.** Extravillious trophoblast (EVTs) cells were transfected with miR-negative control (miR-neg) or miR-210 mimics for 0-72 hours and expression of miR-210 was measured by QPCR. EVT cells were readily transfected to overexpress miR-210 which increasing expression over time. Values are mean ± SEM. Solid line shows results of miR-neg vs miR-210 comparison for each time point, *p<0.0001

**Supplementary Figure S2: miR-210 transfection efficiency of all EVT samples present on the TruSeq Targeted RNA Expression array.** Extravillous trophoblast (EVTs) cells were transfected with miR-negative control (miR-neg) or miR-210 mimics for 24 hours and expression of miR-210 was measured by QPCR prior to isolating RNA for the TruSeq Targeted RNA Expression array. All ten EVT cell lines were readily transfected to overexpress miR-210. Values are mean ± SEM, *p= 0.0037.

**Supplementary Table S1:** Analysis of all predicted or known miR-210 target genes present on the custom TruSeq Targeted RNA Expression Array

| Gene Symbol | Accession | Mean Counts miR-neg | Mean Counts miR-210 | Log2 Fold Change | p-value | Adjusted p-value |
| --- | --- | --- | --- | --- | --- | --- |
| NDUFA4 | NM_002489 | 31817.78 | 13766.96 | -1.25 | 5.63E-27 | 1.40E-24 |
| DIMT1 | NM_014473 | 4352.01 | 2299.21 | -0.88 | 9.02E-20 | 9.26E-18 |
| CNRIP1 | NM_015463 | 3550.17 | 1678.14 | -1.01 | 1.12E-19 | 9.26E-18 |
| SDHD | NM_003002 | 3072.65 | 1577.77 | -0.96 | 2.88E-18 | 1.79E-16 |
| ISCU | NM_014301 | 1980.95 | 919.95 | -1.05 | 4.25E-11 | 2.12E-09 |
| NFIC | NM_205843 | 1156.30 | 539.85 | -1.03 | 2.51E-09 | 1.04E-07 |
| GDE1 | NM_016641 | 933.79 | 434.73 | -1.03 | 7.66E-09 | 2.72E-07 |
| SRP19 | NM_003135 | 1633.79 | 2627.09 | 0.64 | 1.67E-08 | 5.21E-07 |
| ATXN10 | NM_013236 | 5616.47 | 7209.63 | 0.34 | 5.34E-07 | 1.48E-05 |
| SUPT7L | NM_014860 | 536.24 | 854.96 | 0.67 | 4.58E-06 | 1.14E-04 |
| TTC13 | NM_024525 | 548.30 | 292.87 | -0.87 | 6.00E-06 | 1.36E-04 |
| IGF2BP2 | NM_006548 | 1484.60 | 2037.60 | 0.44 | 6.94E-06 | 1.44E-04 |
| STMN1 | NM_203401 | 13504.80 | 17548.96 | 0.36 | 1.48E-05 | 2.83E-04 |
| FLNC | NM_001458 | 2075.21 | 1530.57 | -0.51 | 2.00E-05 | 3.42E-04 |
| SLC7A11 | NM_014331 | 1633.67 | 985.72 | -0.86 | 2.06E-05 | 3.42E-04 |
| SDF2 | NR_045585 | 3417.25 | 2267.40 | -0.65 | 3.42E-05 | 5.32E-04 |
| CD59 | NM_203330 | 19738.27 | 24029.04 | 0.28 | 3.83E-05 | 5.61E-04 |
| PXDC1 | NM_183373 | 2253.37 | 1476.24 | -0.63 | 4.26E-05 | 5.89E-04 |
| RIT1 | NM_006912 | 1698.60 | 2407.20 | 0.49 | 5.56E-05 | 7.28E-04 |
| TNPO1 | NM_153188 | 2070.57 | 1486.24 | -0.47 | 6.10E-05 | 7.59E-04 |
| RPL22 | NM_000983 | 60962.55 | 73405.63 | 0.28 | 8.50E-05 | 1.01E-03 |
| ANKRD13A | NM_033121 | 1648.20 | 1226.47 | -0.45 | 1.12E-04 | 1.26E-03 |
| CELF1 | NM_001172639 | 1908.62 | 1237.84 | -0.56 | 1.28E-04 | 1.38E-03 |
| CENPN | NM_001100625 | 1433.48 | 1939.37 | 0.53 | 1.34E-04 | 1.39E-03 |
| RAB7A | NM_004637 | 6673.94 | 8084.08 | 0.28 | 2.10E-04 | 2.09E-03 |
| KAT6A | NM_001099413 | 709.18 | 401.94 | -0.68 | 3.31E-04 | 3.17E-03 |
| B4GALT5 | NM_004776 | 338.74 | 168.59 | -0.87 | 4.00E-04 | 3.69E-03 |
| NAT14 | NM_020378 | 936.82 | 1365.44 | 0.50 | 5.65E-04 | 5.02E-03 |
| SCARA3 | NM_182826 | 555.14 | 404.76 | -0.67 | 8.47E-04 | 7.27E-03 |
| FCHSD2 | NM_014824 | 630.36 | 376.07 | -0.73 | 8.95E-04 | 7.43E-03 |
| RAB3B | NM_002867 | 970.29 | 604.62 | -0.57 | 1.29E-03 | 0.010 |
| CNP | NM_033133 | 704.83 | 1119.34 | 0.61 | 1.53E-03 | 0.012 |
| SF3B3 | NM_012426 | 1551.77 | 2127.66 | 0.48 | 1.63E-03 | 0.012 |
| FUNDC2 | NM_023934 | 877.27 | 1246.34 | 0.46 | 1.75E-03 | 0.013 |
| LDOC1L | NM_032287 | 2015.11 | 2709.44 | 0.40 | 2.19E-03 | 0.015 |
| AGPAT2 | NM_006412 | 432.25 | 194.12 | -0.86 | 2.24E-03 | 0.015 |
| KIAA0930 | NM_015264 | 314.18 | 180.51 | -0.66 | 3.69E-03 | 0.024 |
| SPRED2 | NM_181784 | 317.38 | 213.02 | -0.58 | 3.72E-03 | 0.024 |
| FGFRL1 | NM_001004358 | 232.25 | 107.28 | -0.84 | 3.96E-03 | 0.025 |
| ZBTB34 | NM_001099270 | 278.69 | 477.28 | 0.65 | 5.22E-03 | 0.032 |
| WDFY2 | NM_052950 | 898.85 | 1337.58 | 0.46 | 5.55E-03 | 0.034 |
| ZNF148 | NM_021964 | 1078.20 | 1527.94 | 0.45 | 5.66E-03 | 0.034 |
| PGAM5 | NM_138575 | 408.51 | 232.70 | -0.71 | 6.61E-03 | 0.038 |
| PTPN21 | NM_007039 | 443.66 | 219.26 | -0.77 | 6.80E-03 | 0.038 |
| MRPL36 | NM_032479 | 3766.42 | 2949.65 | -0.33 | 6.85E-03 | 0.038 |
| PDAP1 | NM_014891 | 2124.65 | 2763.26 | 0.37 | 7.10E-03 | 0.038 |
| LYN | NM_002350 | 142.33 | 341.93 | 0.82 | 7.59E-03 | 0.040 |
| TM4SF18 | NM_138786 | 4.82 | 48.61 | 0.61 | 7.61E-03 | NA |
| KCMF1 | NM_020122 | 1047.88 | 1481.31 | 0.45 | 8.13E-03 | 0.042 |
| SHB | NM_003028 | 571.80 | 857.65 | 0.54 | 8.59E-03 | 0.044 |
| NACC2 | NM_144653 | 1870.87 | 1498.22 | -0.36 | 0.011 | 0.053 |
| RNF170 | NM_030954 | 393.62 | 574.85 | 0.53 | 0.012 | 0.061 |
| PRKCA | NM_002737 | 781.71 | 1053.52 | 0.45 | 0.013 | 0.061 |
| ARRDC4 | NM_183376 | 489.67 | 648.36 | 0.38 | 0.013 | 0.063 |
| CLEC16A | NM_015226 | 904.51 | 1157.08 | 0.31 | 0.014 | 0.065 |
| CSNK1E | NM_152221 | 1645.72 | 1260.63 | -0.37 | 0.015 | 0.066 |
| MEF2D | NM_005920 | 149.41 | 248.37 | 0.58 | 0.015 | 0.068 |
| CORO1C | NM_014325 | 4054.12 | 4561.10 | 0.17 | 0.017 | 0.074 |
| GGNBP2 | NM_024835 | 501.58 | 647.76 | 0.39 | 0.017 | 0.074 |
| CUL3 | NM_003590 | 1202.97 | 1566.14 | 0.31 | 0.018 | 0.074 |
| MTMR9 | NM_015458 | 320.01 | 419.85 | 0.46 | 0.022 | 0.091 |
| ZDHHC20 | NM_153251 | 901.37 | 1249.13 | 0.38 | 0.024 | 0.099 |
| THBS2 | NM_003247 | 3048.96 | 2829.73 | -0.25 | 0.025 | 0.101 |
| TNPO3 | NR_034053 | 907.91 | 711.63 | -0.42 | 0.028 | 0.110 |
| ZNF626 | NM_145297 | 959.65 | 1250.92 | 0.37 | 0.028 | 0.110 |
| SH3BGRL | NM_003022 | 6636.08 | 5749.87 | -0.23 | 0.030 | 0.116 |
| VAMP4 | NR_033704 | 182.45 | 282.21 | 0.51 | 0.032 | 0.122 |
| ARHGEF17 | NM_014786 | 205.65 | 128.25 | -0.60 | 0.033 | NA |
| H2AFY | NM_138610 | 426.44 | 324.05 | -0.42 | 0.033 | 0.124 |
| C16orf72 | NM_014117 | 1207.63 | 1683.55 | 0.36 | 0.035 | 0.129 |
| MID1IP1 | NM_001098790 | 220.63 | 136.54 | -0.56 | 0.038 | NA |
| ADAMTS6 | NM_197941 | 153.46 | 299.98 | 0.61 | 0.039 | 0.141 |
| AKAP2 | NM_001198656 | 337.15 | 235.84 | -0.42 | 0.041 | 0.142 |
| SLC2A4RG | NM_020062 | 626.38 | 830.18 | 0.39 | 0.041 | 0.142 |
| TFDP2 | NM_001178138 | 520.18 | 687.42 | 0.39 | 0.041 | 0.142 |
| LRRC58 | NM_001099678 | 177.56 | 329.83 | 0.60 | 0.044 | 0.149 |
| CHN1 | NM_001822 | 708.40 | 467.73 | -0.58 | 0.045 | 0.151 |
| ZNF526 | NM_133444 | 140.44 | 72.04 | -0.64 | 0.046 | NA |
| ELOVL6 | NM_001130721 | 622.24 | 460.55 | -0.43 | 0.048 | 0.158 |
| C1orf21 | NM_030806 | 494.21 | 640.74 | 0.36 | 0.049 | 0.158 |
| SIPA1L3 | NM_015073 | 431.13 | 282.75 | -0.53 | 0.049 | 0.158 |
| PHF19 | NM_015651 | 1665.77 | 2065.43 | 0.29 | 0.050 | 0.158 |
| EPB41L5 | NM_020909 | 129.57 | 53.32 | -0.64 | 0.051 | NA |
| SIN3B | NM_015260 | 181.06 | 79.79 | -0.64 | 0.052 | NA |
| SMAD4 | NM_005359 | 580.81 | 845.01 | 0.41 | 0.054 | 0.169 |
| TSPAN14 | NM_030927 | 590.61 | 882.11 | 0.34 | 0.054 | 0.169 |
| KLHDC4 | NM_017566 | 105.19 | 239.91 | 0.63 | 0.056 | NA |
| RAB27B | NM_004163 | 94.34 | 25.41 | -0.61 | 0.057 | NA |
| PTBP1 | NM_031990 | 4678.40 | 5357.41 | 0.21 | 0.057 | 0.175 |
| NT5DC1 | NM_152729 | 1412.66 | 1696.19 | 0.27 | 0.059 | 0.180 |
| QKI | NM_006775 | 3215.84 | 4071.23 | 0.28 | 0.061 | 0.182 |
| KIAA0240 | NM_015349 | 337.15 | 203.51 | -0.53 | 0.063 | 0.186 |
| MYOCD | NM_001146312 | 355.30 | 413.19 | 0.52 | 0.064 | 0.186 |
| RBFA | NM_024805 | 488.21 | 666.09 | 0.36 | 0.064 | 0.186 |
| NAA30 | NM_001011713 | 633.95 | 514.44 | -0.32 | 0.068 | 0.194 |
| POLR3A | NM_007055 | 516.40 | 672.23 | 0.34 | 0.069 | 0.195 |
| EHD2 | NM_014601 | 2156.04 | 1814.78 | -0.28 | 0.070 | 0.195 |
| TTC9 | NM_015351 | 3.55 | 27.46 | 0.47 | 0.071 | NA |
| RSU1 | NM_012425 | 5693.37 | 6225.43 | 0.14 | 0.073 | 0.201 |
| OSBPL2 | NM_144498 | 134.68 | 190.06 | 0.42 | 0.079 | NA |
| RCAN1 | NM_203418 | 1195.80 | 1448.91 | 0.32 | 0.080 | 0.218 |
| METTL13 | NM_015935 | 216.22 | 135.67 | -0.48 | 0.082 | 0.222 |
| ZNF512B | NM_020713 | 233.66 | 398.37 | 0.50 | 0.087 | 0.232 |
| GIT2 | NM_057169 | 703.20 | 588.07 | -0.26 | 0.088 | 0.233 |
| FKBP9 | NM_007270 | 789.04 | 678.54 | -0.27 | 0.092 | 0.241 |
| ACTR1A | NM_005736 | 2766.00 | 2446.87 | -0.19 | 0.093 | 0.241 |
| SEC14L1 | NM_001204408 | 739.12 | 605.85 | -0.25 | 0.097 | 0.248 |
| ZNF827 | NM_178835 | 463.89 | 389.70 | -0.38 | 0.099 | 0.251 |
| RMND5A | NM_022780 | 378.55 | 545.93 | 0.45 | 0.100 | 0.251 |
| MXRA7 | NM_001008529 | 686.06 | 880.99 | 0.29 | 0.102 | 0.254 |
| ARMC1 | NM_018120 | 1188.36 | 1032.01 | -0.26 | 0.109 | 0.267 |
| POMT2 | NM_013382 | 123.68 | 88.89 | -0.51 | 0.109 | NA |
| MRPS10 | NM_018141 | 2705.80 | 3185.57 | 0.20 | 0.109 | 0.267 |
| MED20 | NM_004275 | 223.65 | 111.99 | -0.51 | 0.117 | 0.280 |
| NUP50 | NM_153645 | 524.81 | 686.65 | 0.28 | 0.117 | 0.280 |
| ZNF407 | NM_017757 | 70.60 | 103.27 | 0.47 | 0.119 | NA |
| SEMA6A | NM_020796 | 81.28 | 19.93 | -0.46 | 0.121 | NA |
| PMM2 | NM_000303 | 343.07 | 424.38 | 0.25 | 0.122 | 0.290 |
| PIGN | NM_176787 | 181.71 | 284.81 | 0.50 | 0.123 | 0.290 |
| CNKSR2 | NM_014927 | 33.43 | 0.00 | -0.30 | 0.123 | NA |
| CAMTA1 | NM_015215 | 3398.13 | 4065.15 | 0.24 | 0.126 | 0.294 |
| ZNF445 | NM_181489 | 127.60 | 208.80 | 0.46 | 0.127 | NA |
| ARRB1 | NM_004041 | 111.93 | 228.36 | 0.50 | 0.129 | NA |
| TNIP1 | NM_006058 | 1940.18 | 2198.56 | 0.20 | 0.130 | 0.300 |
| ATG7 | NM_006395 | 717.87 | 901.13 | 0.34 | 0.133 | 0.300 |
| CIAPIN1 | NM_020313 | 276.06 | 359.82 | 0.34 | 0.133 | 0.300 |
| SMG5 | NM_015327 | 391.79 | 499.37 | 0.34 | 0.135 | 0.302 |
| MAF | NM_005360 | 114.41 | 104.18 | -0.48 | 0.135 | NA |
| ADAMTS5 | NM_007038 | 580.85 | 471.87 | -0.35 | 0.139 | 0.306 |
| ZMIZ2 | NM_031449 | 263.42 | 185.94 | -0.44 | 0.139 | 0.306 |
| KLF12 | NM_007249 | 105.66 | 164.38 | 0.47 | 0.140 | NA |
| FAM13A | NM_014883 | 61.39 | 136.59 | 0.49 | 0.142 | NA |
| INO80D | NM_017759 | 498.56 | 425.64 | -0.34 | 0.143 | 0.313 |
| TMEM108 | NM_023943 | 83.73 | 161.54 | 0.48 | 0.144 | NA |
| TRIOBP | NM_001039141 | 984.54 | 867.12 | -0.24 | 0.145 | 0.315 |
| TRMT61A | NM_152307 | 157.86 | 257.27 | 0.47 | 0.147 | 0.316 |
| IQSEC1 | NM_001134382 | 147.16 | 77.93 | -0.48 | 0.147 | NA |
| RCAN2 | NM_001251974 | 133.74 | 47.58 | -0.46 | 0.153 | NA |
| UMPS | NR_033437 | 666.25 | 840.13 | 0.29 | 0.155 | 0.330 |
| SLC6A12 | NM_003044 | 2.53 | 0.00 | -0.14 | 0.159 | NA |
| SLCO3A1 | NM_001145044 | 15.17 | 41.91 | 0.39 | 0.161 | NA |
| ISLR2 | NM_001130138 | 20.30 | 0.00 | -0.14 | 0.166 | NA |
| WLS | NM_024911 | 1724.25 | 1446.46 | -0.18 | 0.171 | 0.361 |
| MGRN1 | NM_015246 | 854.51 | 705.56 | -0.24 | 0.173 | 0.363 |
| PRRG2 | NM_000951 | 0.00 | 10.97 | 0.14 | 0.174 | NA |
| EPB41L1 | NM_012156 | 164.74 | 123.95 | -0.38 | 0.175 | NA |
| DAK | NM_015533 | 7.31 | 21.30 | 0.37 | 0.179 | NA |
| UPF1 | NM_002911 | 669.07 | 798.36 | 0.22 | 0.181 | 0.376 |
| ABCA13 | NM_152701 | 1.01 | 0.00 | -0.13 | 0.181 | NA |
| PRMT2 | NM_206962 | 1070.95 | 1256.44 | 0.19 | 0.183 | 0.377 |
| CLDN1 | NM_021101 | 624.36 | 889.50 | 0.39 | 0.186 | 0.379 |
| RRP1B | NM_015056 | 178.34 | 136.74 | -0.33 | 0.187 | NA |
| TRIB1 | NM_025195 | 389.65 | 260.07 | -0.34 | 0.189 | 0.379 |
| N6AMT1 | NM_013240 | 755.08 | 944.26 | 0.28 | 0.189 | 0.379 |
| GPD1L | NM_015141 | 70.43 | 26.51 | -0.37 | 0.195 | NA |
| TCEANC2 | NM_153035 | 130.55 | 79.36 | -0.42 | 0.195 | NA |
| SLC35G1 | NM_153226 | 96.43 | 37.03 | -0.38 | 0.200 | NA |
| E2F3 | NM_001949 | 182.04 | 269.39 | 0.39 | 0.207 | 0.412 |
| FOXD2 | NM_004474 | 18.87 | 2.00 | -0.26 | 0.208 | NA |
| WDR48 | NM_020839 | 323.73 | 251.16 | -0.29 | 0.209 | 0.412 |
| SLC4A11 | NM_001174090 | 10.19 | 0.08 | -0.20 | 0.209 | NA |
| C11orf87 | NM_207645 | 158.70 | 77.14 | -0.39 | 0.213 | NA |
| C12orf59 | NM_153022 | 96.21 | 53.13 | -0.41 | 0.213 | NA |
| ANTXR1 | NM_032208 | 3533.80 | 3811.15 | 0.15 | 0.223 | 0.435 |
| NCOR1 | NM_006311 | 1585.62 | 1357.89 | -0.30 | 0.225 | 0.435 |
| BTN2A1 | NM_078476 | 350.73 | 448.87 | 0.29 | 0.227 | 0.435 |
| ZFAND3 | NM_021943 | 962.93 | 835.75 | -0.21 | 0.227 | 0.435 |
| PRR11 | NM_018304 | 2021.92 | 2292.81 | 0.17 | 0.236 | 0.441 |
| SYNGR2 | NM_004710 | 944.85 | 1230.52 | 0.20 | 0.237 | 0.441 |
| STK24 | NM_003576 | 2341.41 | 2607.46 | 0.14 | 0.238 | 0.441 |
| PDK2 | NM_001199898 | 322.77 | 404.39 | 0.24 | 0.240 | 0.441 |
| NOL12 | NM_024313 | 350.42 | 472.33 | 0.34 | 0.242 | 0.441 |
| SNTB2 | NM_006750 | 603.27 | 486.04 | -0.25 | 0.244 | 0.441 |
| MLLT1 | NM_005934 | 263.75 | 167.39 | -0.38 | 0.244 | 0.441 |
| SMARCA4 | NM_001128849 | 275.33 | 359.55 | 0.31 | 0.244 | 0.441 |
| EPHA2 | NM_004431 | 45.14 | 25.37 | -0.38 | 0.245 | NA |
| WHSC1L1 | NM_023034 | 376.97 | 494.54 | 0.34 | 0.251 | 0.450 |
| PPP1R2 | NM_006241 | 1736.92 | 1554.40 | -0.14 | 0.255 | 0.453 |
| MOCS1 | NM_001075098 | 46.30 | 17.82 | -0.35 | 0.255 | NA |
| MEF2A | NM_001171894 | 587.46 | 692.67 | 0.22 | 0.258 | 0.454 |
| DSG1 | NM_001942 | 0.41 | 0.00 | -0.11 | 0.258 | NA |
| GET4 | NM_015949 | 381.82 | 430.59 | 0.19 | 0.259 | 0.454 |
| TPD52L2 | NM_199360 | 1031.51 | 1200.89 | 0.17 | 0.261 | 0.454 |
| USP6NL | NM_014688 | 122.31 | 90.65 | -0.37 | 0.262 | NA |
| CPEB2 | NM_001177382 | 391.57 | 328.09 | -0.25 | 0.262 | 0.454 |
| C3orf18 | NM_016210 | 26.66 | 77.55 | 0.32 | 0.263 | NA |
| SLC9A8 | NM_015266 | 184.73 | 109.80 | -0.37 | 0.265 | NA |
| ZIC4 | NM_032153 | 35.22 | 15.97 | -0.26 | 0.265 | NA |
| AMMECR1 | NM_001171689 | 94.60 | 72.51 | -0.36 | 0.267 | NA |
| OBSCN | NM_001098623 | 1.89 | 15.67 | 0.15 | 0.271 | NA |
| TOX2 | NM_032883 | 40.65 | 89.94 | 0.32 | 0.271 | NA |
| SLC16A14 | NM_152527 | 23.19 | 10.14 | -0.30 | 0.273 | NA |
| NCAM1 | NM_001242607 | 296.55 | 246.74 | -0.36 | 0.274 | 0.470 |
| C4orf39 | NM_153027 | 24.65 | 10.20 | -0.27 | 0.277 | NA |
| ONECUT2 | NM_004852 | 0.00 | 2.98 | 0.11 | 0.278 | NA |
| PREPL | NM_001171606 | 1590.67 | 1519.05 | -0.22 | 0.278 | 0.475 |
| GFRA1 | NM_005264 | 293.76 | 307.49 | 0.31 | 0.281 | 0.475 |
| DNAJC6 | NM_014787 | 182.63 | 274.17 | 0.34 | 0.282 | 0.475 |
| ICMT | NM_012405 | 1691.97 | 1846.74 | 0.13 | 0.285 | 0.477 |
| WDR7 | NM_015285 | 157.75 | 97.63 | -0.35 | 0.286 | NA |
| RCN3 | NM_020650 | 1866.17 | 2131.67 | 0.14 | 0.287 | 0.477 |
| KCTD16 | NM_020768 | 160.78 | 93.35 | -0.34 | 0.293 | NA |
| FBXL19 | NM_001099784 | 243.62 | 334.29 | 0.31 | 0.305 | 0.503 |
| DLX1 | NM_178120 | 97.20 | 42.60 | -0.31 | 0.315 | NA |
| ADAT3 | NM_138422 | 47.42 | 86.74 | 0.32 | 0.320 | NA |
| ARHGAP35 | NM_004491 | 2793.53 | 2653.38 | -0.11 | 0.325 | 0.532 |
| PPP1CB | NM_206876 | 4005.11 | 3562.16 | -0.22 | 0.328 | 0.534 |
| PSAP | NM_001042465 | 2503.12 | 2268.79 | -0.11 | 0.332 | 0.537 |
| YAF2 | NR_034000 | 755.74 | 889.09 | 0.16 | 0.344 | 0.553 |
| DSC3 | NM_024423 | 80.62 | 40.01 | -0.29 | 0.346 | NA |
| KIAA1161 | NM_020702 | 18.93 | 6.66 | -0.25 | 0.347 | NA |
| SAMD12 | NM_001101676 | 151.54 | 86.72 | -0.28 | 0.351 | NA |
| COG6 | NR_026745 | 1259.30 | 1161.34 | -0.13 | 0.359 | 0.573 |
| C19orf25 | NM_152482 | 245.76 | 286.71 | 0.23 | 0.362 | 0.574 |
| INPP5A | NM_005539 | 104.07 | 88.15 | -0.28 | 0.370 | NA |
| MFSD4 | NM_181644 | 6.55 | 0.13 | -0.12 | 0.373 | NA |
| NTN1 | NM_004822 | 1.55 | 7.67 | 0.09 | 0.373 | NA |
| HOXC6 | NM_153693 | 36.49 | 18.45 | -0.24 | 0.376 | NA |
| NFAT5 | NM_138714 | 1460.68 | 1714.07 | 0.13 | 0.381 | 0.599 |
| TSTD2 | NM_139246 | 112.23 | 126.99 | 0.24 | 0.382 | NA |
| PPM1F | NM_014634 | 383.47 | 308.38 | -0.18 | 0.383 | 0.599 |
| TSPAN10 | NM_031945 | 14.95 | 2.76 | -0.18 | 0.385 | NA |
| KY | NM_178554 | 6.15 | 0.00 | -0.09 | 0.386 | NA |
| ZXDB | NM_007157 | 255.25 | 188.96 | -0.25 | 0.393 | 0.611 |
| PPP1R3B | NM_001201329 | 0.52 | 2.68 | 0.09 | 0.399 | NA |
| ZNF555 | NM_152791 | 42.82 | 65.60 | 0.27 | 0.399 | NA |
| DYRK2 | NM_006482 | 274.86 | 363.00 | 0.23 | 0.400 | 0.615 |
| LRRC8A | NM_019594 | 197.89 | 149.75 | -0.26 | 0.402 | 0.615 |
| ATXN1 | NM_000332 | 324.43 | 392.18 | 0.24 | 0.403 | 0.615 |
| C14orf119 | NM_017924 | 29.31 | 67.30 | 0.22 | 0.406 | NA |
| VPS13C | NM_020821 | 143.51 | 200.37 | 0.26 | 0.408 | NA |
| KIAA0141 | NM_001142603 | 124.56 | 189.86 | 0.27 | 0.409 | NA |
| CCNL2 | NM_030937 | 526.04 | 581.20 | 0.13 | 0.411 | 0.624 |
| SLC35B4 | NM_032826 | 611.24 | 710.81 | 0.17 | 0.416 | 0.628 |
| THSD7A | NM_015204 | 5.03 | 0.00 | -0.08 | 0.420 | NA |
| MX1 | NM_001144925 | 35.08 | 38.35 | 0.23 | 0.422 | NA |
| TMEM241 | NM_032933 | 160.29 | 117.74 | -0.25 | 0.424 | NA |
| SUSD4 | NM_017982 | 3.58 | 0.00 | -0.08 | 0.431 | NA |
| MAPK1 | NM_002745 | 1601.33 | 1533.38 | -0.10 | 0.436 | 0.651 |
| FKBP1B | NM_054033 | 115.23 | 67.95 | -0.24 | 0.436 | NA |
| SLC4A4 | NM_001098484 | 277.65 | 240.44 | -0.19 | 0.436 | 0.651 |
| MYH11 | NM_001040113 | 17.27 | 3.46 | -0.08 | 0.438 | NA |
| SLC25A26 | NM_173471 | 430.29 | 472.42 | 0.17 | 0.440 | 0.652 |
| CCNG2 | NM_004354 | 903.20 | 930.38 | 0.11 | 0.448 | 0.661 |
| GCNT1 | NM_001490 | 140.28 | 119.25 | -0.24 | 0.449 | NA |
| SEC14L2 | NM_012429 | 110.47 | 75.67 | -0.25 | 0.453 | NA |
| FAM116A | NM_152678 | 155.32 | 125.40 | -0.24 | 0.458 | NA |
| ZNF155 | NM_198089 | 493.93 | 550.22 | 0.17 | 0.461 | 0.675 |
| ABCC1 | NM_004996 | 590.32 | 540.42 | -0.13 | 0.464 | 0.676 |
| NHS | NM_001136024 | 60.62 | 98.64 | 0.23 | 0.468 | NA |
| LEPROTL1 | NM_015344 | 955.77 | 878.46 | -0.11 | 0.473 | 0.685 |
| EFNA3 | NM_004952 | 2.99 | 0.00 | -0.07 | 0.474 | NA |
| PPM1K | NM_152542 | 82.00 | 46.97 | -0.21 | 0.476 | NA |
| NEURL1B | NM_001142651 | 196.82 | 273.17 | 0.21 | 0.479 | NA |
| EIF2C4 | NM_017629 | 66.70 | 83.74 | 0.23 | 0.479 | NA |
| POLR3F | NM_006466 | 392.30 | 419.59 | 0.17 | 0.480 | 0.690 |
| STAG3L1 | NR_040583 | 451.01 | 523.52 | 0.13 | 0.486 | 0.695 |
| PPM1H | NM_020700 | 88.49 | 81.80 | -0.22 | 0.489 | NA |
| RUNX3 | NM_001031680 | 5.14 | 0.49 | -0.07 | 0.494 | NA |
| RBFOX2 | NM_001082579 | 2225.91 | 2290.55 | 0.06 | 0.495 | 0.705 |
| MNT | NM_020310 | 132.62 | 116.26 | -0.20 | 0.498 | NA |
| TNPO2 | NM_001136195 | 913.53 | 1054.32 | 0.13 | 0.499 | 0.707 |
| B3GALT5 | NM_033171 | 0.00 | 1.78 | 0.07 | 0.503 | NA |
| DCAF5 | NM_003861 | 84.54 | 106.13 | 0.21 | 0.506 | NA |
| GEMIN8 | NM_017856 | 239.56 | 266.83 | 0.16 | 0.514 | 0.723 |
| ITGB8 | NM_002214 | 20.63 | 29.09 | 0.20 | 0.519 | NA |
| TET2 | NM_001127208 | 100.25 | 78.65 | -0.21 | 0.522 | NA |
| HOXA1 | NM_153620 | 8.15 | 2.47 | -0.14 | 0.522 | NA |
| TMEM40 | NM_018306 | 11.12 | 5.94 | -0.14 | 0.527 | NA |
| PRLR | NM_001204316 | 11.53 | 16.88 | 0.14 | 0.528 | NA |
| PITHD1 | NM_020362 | 688.95 | 735.21 | 0.10 | 0.529 | 0.739 |
| CHRM3 | NM_000740 | 0.00 | 8.36 | 0.07 | 0.530 | NA |
| BTBD9 | NM_052893 | 121.83 | 94.29 | -0.20 | 0.532 | NA |
| HDAC4 | NM_006037 | 2.51 | 0.00 | -0.06 | 0.534 | NA |
| ZNF678 | NM_178549 | 10.70 | 5.99 | -0.13 | 0.535 | NA |
| AGPAT6 | NM_178819 | 120.66 | 145.31 | 0.18 | 0.536 | NA |
| NPTX1 | NM_002522 | 27.61 | 43.70 | 0.19 | 0.538 | NA |
| PLEKHM3 | NM_001080475 | 79.71 | 65.42 | -0.20 | 0.540 | NA |
| HIATL1 | NM_032558 | 600.95 | 525.64 | -0.12 | 0.546 | 0.759 |
| CYGB | NM_134268 | 13.51 | 24.54 | 0.15 | 0.549 | NA |
| ZNF462 | NM_021224 | 222.31 | 185.62 | -0.18 | 0.553 | 0.765 |
| RNF212 | NM_194439 | 43.20 | 28.07 | -0.17 | 0.555 | NA |
| PPP1R9A | NM_001166160 | 3.07 | 0.00 | -0.06 | 0.556 | NA |
| ANAPC7 | NM_016238 | 1099.95 | 1260.93 | 0.09 | 0.557 | 0.766 |
| LRFN1 | NM_020862 | 10.64 | 3.35 | -0.08 | 0.563 | NA |
| ADCY1 | NM_021116 | 2.97 | 8.09 | 0.10 | 0.565 | NA |
| MEF2C | NM_001193347 | 180.62 | 182.10 | -0.18 | 0.568 | NA |
| C15orf52 | NM_207380 | 110.09 | 85.40 | -0.17 | 0.570 | NA |
| ELFN2 | NM_052906 | 3.74 | 0.00 | -0.06 | 0.572 | NA |
| KIAA1143 | NM_020696 | 1079.21 | 1193.54 | 0.10 | 0.572 | 0.783 |
| ALPK1 | NM_025144 | 19.14 | 12.20 | -0.16 | 0.575 | NA |
| APOBEC3F | NM_145298 | 97.64 | 88.70 | -0.17 | 0.582 | NA |
| ACVR1B | NM_020328 | 59.58 | 92.27 | 0.16 | 0.583 | NA |
| CEND1 | NM_016564 | 17.84 | 7.67 | -0.11 | 0.583 | NA |
| NR3C2 | NM_000901 | 7.34 | 0.00 | -0.06 | 0.586 | NA |
| NR1D2 | NM_005126 | 288.08 | 243.48 | -0.16 | 0.586 | 0.798 |
| ALDH5A1 | NM_170740 | 79.91 | 96.33 | 0.18 | 0.587 | NA |
| ENOX2 | NM_182314 | 144.47 | 118.62 | -0.16 | 0.589 | NA |
| ANKS3 | NM_133450 | 28.01 | 20.85 | -0.15 | 0.590 | NA |
| INF2 | NM_022489 | 174.06 | 201.68 | 0.13 | 0.591 | NA |
| SIN3A | NM_015477 | 72.57 | 88.51 | 0.16 | 0.593 | NA |
| OTUB2 | NM_023112 | 41.26 | 75.89 | 0.14 | 0.594 | NA |
| SEC24B | NM_006323 | 123.73 | 149.81 | 0.15 | 0.595 | NA |
| KPNA1 | NR_026698 | 235.13 | 257.99 | 0.11 | 0.595 | 0.805 |
| ANKRD24 | NM_133475 | 1.81 | 5.72 | 0.05 | 0.597 | NA |
| FAM159B | NM_001164442 | 5.30 | 1.97 | -0.05 | 0.600 | NA |
| NKAIN2 | NM_001040214 | 0.52 | 0.00 | -0.05 | 0.602 | NA |
| TSPAN31 | NM_005981 | 252.40 | 290.44 | 0.12 | 0.602 | 0.809 |
| DENND5B | NM_144973 | 191.76 | 218.02 | 0.17 | 0.604 | 0.809 |
| C2CD2 | NM_015500 | 19.39 | 35.58 | 0.14 | 0.605 | NA |
| DOT1L | NM_032482 | 154.48 | 193.41 | 0.17 | 0.606 | NA |
| KAT6B | NM_012330 | 245.24 | 245.01 | -0.14 | 0.611 | 0.810 |
| OXTR | NM_000916 | 667.16 | 548.75 | -0.09 | 0.612 | 0.810 |
| CSF2RB | NM_000395 | 55.98 | 56.69 | -0.16 | 0.618 | NA |
| BNC2 | NM_017637 | 136.55 | 113.06 | -0.16 | 0.621 | NA |
| APOBEC3D | NM_152426 | 4.68 | 10.04 | 0.09 | 0.624 | NA |
| FANCF | NM_022725 | 49.64 | 72.24 | 0.15 | 0.626 | NA |
| MLL | NM_005933 | 285.11 | 261.48 | -0.15 | 0.629 | 0.829 |
| C12orf34 | NM_032829 | 44.71 | 37.07 | -0.14 | 0.631 | NA |
| MID2 | NM_052817 | 302.00 | 269.71 | -0.15 | 0.633 | 0.830 |
| MAB21L3 | NM_152367 | 3.85 | 0.00 | -0.05 | 0.638 | NA |
| SOX15 | NM_006942 | 25.87 | 19.79 | -0.13 | 0.638 | NA |
| MXD4 | NM_006454 | 1429.18 | 1499.77 | 0.08 | 0.638 | 0.832 |
| FAM102A | NM_001035254 | 331.31 | 388.54 | 0.13 | 0.643 | 0.833 |
| BMP6 | NM_001718 | 10.49 | 21.43 | 0.10 | 0.645 | NA |
| KIF13B | NM_015254 | 48.78 | 68.55 | 0.14 | 0.646 | NA |
| LIN52 | NM_001024674 | 164.33 | 183.53 | 0.11 | 0.649 | NA |
| TNS1 | NM_022648 | 1441.67 | 1421.92 | -0.07 | 0.659 | 0.844 |
| ZNF841 | NM_001136499 | 264.37 | 289.38 | -0.12 | 0.662 | 0.844 |
| CORO2B | NM_006091 | 444.58 | 385.82 | -0.14 | 0.663 | 0.844 |
| BTN2A2 | NM_006995 | 242.21 | 265.45 | 0.13 | 0.666 | 0.844 |
| RAB31 | NM_006868 | 561.47 | 674.25 | 0.10 | 0.668 | 0.844 |
| ZHX2 | NM_014943 | 111.70 | 108.63 | -0.14 | 0.669 | NA |
| KLHL31 | NM_001003760 | 0.05 | 0.25 | 0.04 | 0.672 | NA |
| ERP27 | NM_152321 | 2.24 | 0.00 | -0.04 | 0.678 | NA |
| TECPR2 | NM_014844 | 201.63 | 251.10 | 0.13 | 0.679 | 0.854 |
| IP6K3 | NM_001142883 | 2.30 | 6.10 | 0.04 | 0.680 | NA |
| FOXN3 | NM_001085471 | 1591.85 | 1627.01 | -0.07 | 0.684 | 0.855 |
| CTTN | NM_001184740 | 1690.10 | 1772.65 | 0.05 | 0.688 | 0.855 |
| ANO2 | NM_020373 | 2.32 | 0.00 | -0.04 | 0.689 | NA |
| HIVEP2 | NM_006734 | 840.70 | 792.20 | -0.08 | 0.690 | 0.855 |
| SEMA5B | NR_046079 | 0.36 | 0.00 | -0.04 | 0.690 | NA |
| TYW5 | NR_004862 | 173.42 | 202.79 | 0.12 | 0.693 | NA |
| TMEM35 | NM_021637 | 112.35 | 126.23 | -0.13 | 0.694 | NA |
| PDE10A | NR_045597 | 28.40 | 35.86 | 0.12 | 0.694 | NA |
| GJD3 | NM_152219 | 19.59 | 22.14 | 0.12 | 0.697 | NA |
| KCTD11 | NM_001002914 | 13.05 | 7.07 | -0.10 | 0.699 | NA |
| PLEKHG4B | NM_052909 | 19.96 | 16.88 | -0.09 | 0.711 | NA |
| CHRNB1 | NM_000747 | 9.13 | 5.79 | -0.08 | 0.718 | NA |
| SUZ12 | NM_015355 | 499.25 | 478.13 | -0.08 | 0.718 | 0.885 |
| GRIN2A | NM_001134408 | 19.27 | 4.25 | -0.04 | 0.720 | NA |
| LMBR1 | NM_022458 | 410.84 | 458.92 | 0.08 | 0.723 | 0.886 |
| GOLPH3 | NM_022130 | 1321.10 | 1335.29 | -0.07 | 0.729 | 0.890 |
| SYNPO2 | NM_133477 | 24.01 | 21.04 | -0.09 | 0.732 | NA |
| C4orf44 | NM_001042690 | 0.00 | 0.13 | 0.03 | 0.732 | NA |
| ADCY5 | NM_183357 | 4.51 | 8.63 | 0.06 | 0.735 | NA |
| ADARB1 | NR_027673 | 627.84 | 509.15 | -0.08 | 0.740 | 0.891 |
| CTIF | NM_001142397 | 238.67 | 258.74 | 0.08 | 0.742 | 0.891 |
| POU2F2 | NM_002698 | 192.72 | 244.18 | 0.11 | 0.744 | 0.891 |
| APBA2 | NM_005503 | 121.06 | 158.96 | 0.11 | 0.747 | NA |
| KIAA1755 | NM_001029864 | 0.93 | 3.84 | 0.04 | 0.747 | NA |
| WDFY1 | NM_020830 | 1203.70 | 1218.21 | -0.05 | 0.748 | 0.891 |
| STXBP5L | NM_014980 | 4.85 | 2.86 | -0.03 | 0.750 | NA |
| KIAA1549 | NM_020910 | 20.40 | 26.73 | 0.09 | 0.753 | NA |
| FAM125B | NM_033446 | 198.16 | 173.88 | -0.10 | 0.754 | 0.891 |
| WIPF3 | NM_001080529 | 16.00 | 10.40 | -0.05 | 0.754 | NA |
| PDCD1LG2 | NM_025239 | 1390.59 | 1332.51 | -0.05 | 0.754 | 0.891 |
| ZNF516 | NM_014643 | 253.02 | 217.52 | 0.10 | 0.755 | 0.891 |
| PALM2 | NM_053016 | 151.96 | 156.66 | 0.09 | 0.757 | NA |
| ARL3 | NM_004311 | 1432.87 | 1487.40 | 0.04 | 0.759 | 0.892 |
| ANO4 | NM_178826 | 136.82 | 76.53 | -0.09 | 0.761 | NA |
| PTPRO | NM_030667 | 3.63 | 1.58 | -0.04 | 0.764 | NA |
| PEX10 | NM_153818 | 37.48 | 34.71 | -0.08 | 0.770 | NA |
| SLC29A2 | NM_001532 | 1.16 | 0.00 | -0.03 | 0.771 | NA |
| RGS5 | NM_001254748 | 712.93 | 589.98 | -0.09 | 0.777 | 0.906 |
| PDE3A | NM_000921 | 196.98 | 259.03 | -0.09 | 0.779 | 0.906 |
| RUNX1T1 | NM_001198625 | 142.91 | 131.47 | -0.09 | 0.779 | NA |
| SORBS1 | NM_001034955 | 67.62 | 77.56 | 0.09 | 0.780 | NA |
| TMEM132D | NM_133448 | 34.58 | 34.42 | -0.08 | 0.784 | NA |
| TPPP | NM_007030 | 7.11 | 11.10 | 0.03 | 0.784 | NA |
| ZFPM1 | NM_153813 | 1.61 | 4.94 | 0.04 | 0.785 | NA |
| TMEM204 | NM_001256541 | 294.07 | 348.28 | 0.09 | 0.789 | 0.914 |
| BDNF | NM_001143814 | 36.72 | 32.47 | -0.07 | 0.795 | NA |
| ADCY7 | NM_001114 | 27.06 | 22.00 | -0.05 | 0.797 | NA |
| FBXL16 | NM_153350 | 4.61 | 9.16 | 0.05 | 0.799 | NA |
| NDRG4 | NM_022910 | 73.86 | 84.86 | 0.08 | 0.800 | NA |
| TBC1D30 | NM_015279 | 3.05 | 0.00 | -0.03 | 0.801 | NA |
| RHOQ | NM_012249 | 1626.78 | 1629.87 | -0.03 | 0.803 | 0.925 |
| VMP1 | NM_030938 | 4371.26 | 4387.51 | 0.02 | 0.808 | 0.926 |
| PRPF38B | NR_037185 | 1327.62 | 1463.48 | 0.04 | 0.811 | 0.926 |
| CORO6 | NM_032854 | 8.61 | 11.51 | 0.05 | 0.812 | NA |
| GNAT1 | NM_144499 | 83.85 | 102.84 | 0.07 | 0.813 | NA |
| ABCD1 | NM_000033 | 24.96 | 36.40 | 0.07 | 0.813 | NA |
| ST6GAL2 | NM_032528 | 81.55 | 156.62 | 0.07 | 0.815 | NA |
| TFRC | NM_003234 | 2974.38 | 3026.55 | -0.03 | 0.818 | 0.927 |
| DEAF1 | NM_021008 | 335.46 | 307.23 | -0.07 | 0.821 | 0.927 |
| ZNF805 | NM_001145078 | 0.63 | 1.86 | 0.03 | 0.821 | NA |
| CD22 | NM_001771 | 0.00 | 1.13 | 0.02 | 0.822 | NA |
| MCM8 | NM_182802 | 230.87 | 230.66 | 0.06 | 0.825 | 0.927 |
| MLL2 | NM_003482 | 100.42 | 100.19 | 0.06 | 0.825 | NA |
| HPSE2 | NM_001166246 | 0.00 | 0.99 | 0.02 | 0.826 | NA |
| PDZD4 | NM_032512 | 1.89 | 0.00 | -0.03 | 0.833 | NA |
| CELF2 | NM_006561 | 256.58 | 213.83 | -0.07 | 0.835 | 0.927 |
| IGF2 | NM_000612 | 354.29 | 383.61 | 0.06 | 0.835 | 0.927 |
| TOR1A | NM_000113 | 1386.08 | 1384.50 | -0.03 | 0.837 | 0.927 |
| HIF3A | NM_152795 | 0.53 | 0.00 | -0.02 | 0.837 | NA |
| NRP2 | NM_201266 | 243.70 | 252.31 | -0.06 | 0.838 | 0.927 |
| PCYT1B | NM_001163265 | 0.32 | 0.00 | -0.02 | 0.839 | NA |
| ATP11A | NM_015205 | 34.32 | 28.82 | -0.06 | 0.841 | NA |
| FAM73B | NM_032809 | 108.97 | 104.69 | -0.07 | 0.841 | NA |
| CLIP2 | NM_003388 | 40.56 | 53.88 | 0.06 | 0.842 | NA |
| SLC5A3 | NM_006933 | 165.93 | 193.46 | 0.06 | 0.843 | 0.929 |
| KIAA0664 | NM_015229 | 3.65 | 4.96 | 0.02 | 0.845 | NA |
| AFF2 | NM_002025 | 2.53 | 4.42 | 0.02 | 0.848 | NA |
| BDKRB2 | NM_000623 | 119.68 | 111.46 | -0.06 | 0.849 | NA |
| DICER1 | NM_030621 | 512.20 | 533.68 | 0.04 | 0.850 | 0.930 |
| LURAP1L | NM_203403 | 255.80 | 263.16 | -0.05 | 0.858 | 0.930 |
| CSF1 | NM_172212 | 676.70 | 711.65 | 0.05 | 0.860 | 0.930 |
| ESCO2 | NM_001017420 | 227.57 | 252.57 | 0.05 | 0.861 | 0.930 |
| RAP2B | NM_002886 | 45.45 | 52.33 | 0.05 | 0.862 | NA |
| TPMT | NM_000367 | 709.45 | 698.26 | -0.03 | 0.865 | 0.930 |
| TNRC6B | NM_001024843 | 308.66 | 356.20 | 0.05 | 0.867 | 0.930 |
| AKAP7 | NM_004842 | 34.38 | 30.95 | -0.04 | 0.871 | NA |
| KIF5A | NM_004984 | 5.19 | 4.47 | -0.02 | 0.872 | NA |
| FAM124A | NM_145019 | 43.14 | 44.04 | -0.04 | 0.877 | NA |
| INSIG1 | NM_005542 | 2693.24 | 2414.41 | -0.02 | 0.879 | 0.939 |
| USF1 | NM_207005 | 608.33 | 676.93 | 0.04 | 0.886 | 0.940 |
| TOM1L2 | NM_001082968 | 845.28 | 837.47 | -0.02 | 0.887 | 0.940 |
| SS18L1 | NM_198935 | 73.64 | 66.63 | -0.04 | 0.890 | NA |
| WHSC1 | NM_133330 | 956.73 | 1022.44 | -0.02 | 0.899 | 0.948 |
| LPP | NM_005578 | 1843.83 | 1863.82 | 0.02 | 0.902 | 0.948 |
| C10orf114 | NM_001010911 | 88.18 | 79.15 | -0.04 | 0.907 | NA |
| ARHGAP33 | NM_052948 | 29.72 | 25.47 | -0.03 | 0.910 | NA |
| ZFHX2 | NM_033400 | 24.66 | 22.31 | -0.03 | 0.910 | NA |
| SAMD4A | NM_015589 | 258.28 | 247.39 | -0.03 | 0.916 | 0.958 |
| RAB9B | NM_016370 | 13.63 | 15.76 | 0.02 | 0.918 | NA |
| RFX7 | NM_022841 | 402.65 | 378.40 | -0.03 | 0.919 | 0.958 |
| KCNA3 | NM_002232 | 4.13 | 5.50 | 0.01 | 0.924 | NA |
| CACNG8 | NM_031895 | 2.53 | 2.13 | -0.01 | 0.925 | NA |
| FBXO31 | NR_024568 | 58.47 | 51.44 | -0.03 | 0.925 | NA |
| WDR37 | NM_014023 | 197.88 | 195.81 | -0.02 | 0.925 | NA |
| CACNA1A | NM_023035 | 35.68 | 49.15 | -0.03 | 0.925 | NA |
| CHST1 | NM_003654 | 10.27 | 9.37 | -0.01 | 0.930 | NA |
| MITF | NM_198177 | 584.95 | 665.36 | -0.03 | 0.930 | 0.965 |
| RAPH1 | NM_203365 | 140.92 | 139.09 | -0.02 | 0.935 | NA |
| CCDC68 | NM_025214 | 71.83 | 87.36 | -0.03 | 0.935 | NA |
| TMEM102 | NM_178518 | 33.43 | 39.44 | 0.02 | 0.938 | NA |
| STAT6 | NM_003153 | 773.96 | 699.15 | -0.02 | 0.939 | 0.967 |
| MAML3 | NM_018717 | 82.24 | 73.40 | -0.02 | 0.940 | NA |
| DHX58 | NM_024119 | 7.35 | 8.29 | 0.01 | 0.940 | NA |
| SLC38A1 | NM_030674 | 1559.53 | 1559.01 | -0.01 | 0.944 | 0.967 |
| SLC6A6 | NM_003043 | 488.10 | 469.52 | 0.02 | 0.945 | 0.967 |
| CACNA1C | NM_199460 | 24.79 | 27.31 | 0.02 | 0.947 | NA |
| HMGCS1 | NM_001098272 | 282.43 | 336.07 | -0.02 | 0.949 | 0.967 |
| SEPT8 | NM_015146 | 1623.28 | 1607.18 | -0.01 | 0.951 | 0.967 |
| HOXA9 | NM_152739 | 88.99 | 78.30 | 0.02 | 0.955 | NA |
| SYNGAP1 | NM_006772_1 | 18.64 | 20.27 | 0.01 | 0.959 | NA |
| KLHL24 | NM_017644 | 63.75 | 73.67 | 0.02 | 0.962 | NA |
| BAZ2B | NM_013450 | 406.89 | 454.46 | 0.01 | 0.963 | 0.974 |
| VPS37D | NM_001077621 | 85.34 | 86.61 | 0.01 | 0.967 | NA |
| IL17D | NM_138284 | 23.28 | 20.41 | 0.01 | 0.968 | NA |
| ZNF449 | NM_152695 | 50.29 | 45.72 | -0.01 | 0.970 | NA |
| TSPAN17 | NM_130465 | 824.88 | 860.71 | -0.01 | 0.970 | 0.974 |
| COX10 | NM_001303 | 747.90 | 749.83 | -0.01 | 0.970 | 0.974 |
| NFIX | NM_002501 | 120.94 | 118.91 | 0.01 | 0.971 | NA |
| UCP3 | NM_003356 | 0.83 | 0.75 | 0.00 | 0.983 | NA |
| SLITRK1 | NM_052910 | 0.00 | 0.10 | 0.00 | 0.985 | NA |
| NMNAT2 | NM_170706 | 84.65 | 88.17 | -0.01 | 0.985 | NA |
| ZNF618 | NM_133374 | 189.33 | 196.39 | 0.00 | 0.988 | NA |
| GNG7 | NM_052847 | 5.29 | 7.57 | 0.00 | 0.988 | NA |
| RGMA | NM_020211 | 1.14 | 1.37 | 0.00 | 0.988 | NA |
| ACVR1C | NM_145259 | 10.19 | 10.50 | 0.00 | 0.991 | NA |
| MBTD1 | NM_017643 | 17.77 | 17.93 | 0.00 | 0.997 | NA |
| F7 | NM_000131 | 5.92 | 6.47 | 0.00 | 0.997 | NA |
| MDGA1 | NM_153487 | 129.70 | 136.00 | 0.00 | 0.999 | NA |
| FTSJ2 | NM_013393 | 475.83 | 465.47 | 0.00 | 1.000 | 1.000 |
| BAIAP3 | NM_003933 | No sequencing reads – not detected in sample | | | | |
| C17orf51 | NM_001113434 | No sequencing reads – not detected in sample | | | | |
| C1orf116 | NM_023938 | No sequencing reads – not detected in sample | | | | |
| CLEC2L | NM_001080511 | No sequencing reads – not detected in sample | | | | |
| CPLX2 | NM_006650 | No sequencing reads – not detected in sample | | | | |
| EVL | NM_016337 | No sequencing reads – not detected in sample | | | | |
| FAM123C | NM_001105194 | No sequencing reads – not detected in sample | | | | |
| FOXP3 | NM_014009 | No sequencing reads – not detected in sample | | | | |
| IGLON5 | NM_001101372 | No sequencing reads – not detected in sample | | | | |
| IL16 | NM_001172128 | No sequencing reads – not detected in sample | | | | |
| INHBB | NM_002193 | No sequencing reads – not detected in sample | | | | |
| KCNK3 | NM_002246 | No sequencing reads – not detected in sample | | | | |
| PAX6 | NM_001604 | No sequencing reads – not detected in sample | | | | |
| PIK3R5 | NM_014308 | No sequencing reads – not detected in sample | | | | |
| SHE | NM_001010846 | No sequencing reads – not detected in sample | | | | |
| SLC1A2 | NM_001252652 | No sequencing reads – not detected in sample | | | | |
| TIGIT | NM_173799 | No sequencing reads – not detected in sample | | | | |
| TLX3 | NM_021025 | No sequencing reads – not detected in sample | | | | |
| AIFM3 | NM_144704 | No sequencing reads – not detected in sample | | | | |
| AQP4 | NM_001650 | No sequencing reads – not detected in sample | | | | |
| ASCL1 | NM_004316 | No sequencing reads – not detected in sample | | | | |
| ATP2B3 | NM_021949 | No sequencing reads – not detected in sample | | | | |
| BTK | NM_000061 | No sequencing reads – not detected in sample | | | | |
| C1orf212 | NM_001164824 | No sequencing reads – not detected in sample | | | | |
| C8orf86 | NM_207412 | No sequencing reads – not detected in sample | | | | |
| CACNA2D2 | NM_001174051 | No sequencing reads – not detected in sample | | | | |
| CACNG2 | NM_006078 | No sequencing reads – not detected in sample | | | | |
| CAMK2B | NM_001220 | No sequencing reads – not detected in sample | | | | |
| CAPN9 | NM_006615 | No sequencing reads – not detected in sample | | | | |
| CCBP2 | NM_001296 | No sequencing reads – not detected in sample | | | | |
| CCKBR | NM_176875 | No sequencing reads – not detected in sample | | | | |
| CNTNAP5 | NM_130773 | No sequencing reads – not detected in sample | | | | |
| CR1 | NM_000651 | No sequencing reads – not detected in sample | | | | |
| CREB3L3 | NM_032607 | No sequencing reads – not detected in sample | | | | |
| DRD5 | NM_000798 | No sequencing reads – not detected in sample | | | | |
| DTX1 | NM_004416 | No sequencing reads – not detected in sample | | | | |
| FAM196A | NM_001039762 | No sequencing reads – not detected in sample | | | | |
| FGD4 | NM_139241 | No sequencing reads – not detected in sample | | | | |
| GHSR | NM_198407 | No sequencing reads – not detected in sample | | | | |
| GIMAP1 | NM_130759 | No sequencing reads – not detected in sample | | | | |
| GNA15 | NM_002068 | No sequencing reads – not detected in sample | | | | |
| GPR123 | NM_001083909 | No sequencing reads – not detected in sample | | | | |
| GPR17 | NM_001161415 | No sequencing reads – not detected in sample | | | | |
| GRK1 | NM_002929 | No sequencing reads – not detected in sample | | | | |
| GRM6 | NM_000843 | No sequencing reads – not detected in sample | | | | |
| GUSB | NM_000181 | No sequencing reads – not detected in sample | | | | |
| HMP19 | NM_015980 | No sequencing reads – not detected in sample | | | | |
| INPP5D | NM_005541 | No sequencing reads – not detected in sample | | | | |
| IPO5 | NM_002271 | No sequencing reads – not detected in sample | | | | |
| KAAG1 | NM_181337 | No sequencing reads – not detected in sample | | | | |
| KCNK10 | NM_138318 | No sequencing reads – not detected in sample | | | | |
| KCNK5 | NM_003740 | No sequencing reads – not detected in sample | | | | |
| KCNQ4 | NM_004700 | No sequencing reads – not detected in sample | | | | |
| KIF1A | NM_001244008 | No sequencing reads – not detected in sample | | | | |
| KLB | NM_175737 | No sequencing reads – not detected in sample | | | | |
| LAIR1 | NM_002287 | No sequencing reads – not detected in sample | | | | |
| LDLRAD1 | NM_001010978 | No sequencing reads – not detected in sample | | | | |
| LHFPL4 | NM_198560 | No sequencing reads – not detected in sample | | | | |
| LMAN1L | NM_021819 | No sequencing reads – not detected in sample | | | | |
| LMX1B | NM_002316 | No sequencing reads – not detected in sample | | | | |
| LTA | NM_000595_3 | No sequencing reads – not detected in sample | | | | |
| MARVELD3 | NM_052858 | No sequencing reads – not detected in sample | | | | |
| MLC1 | NM_139202 | No sequencing reads – not detected in sample | | | | |
| MPEG1 | NM_001039396 | No sequencing reads – not detected in sample | | | | |
| MYT1L | NM_015025 | No sequencing reads – not detected in sample | | | | |
| NEUROD2 | NM_006160 | No sequencing reads – not detected in sample | | | | |
| NGFR | NM_002507 | No sequencing reads – not detected in sample | | | | |
| NUP210 | NM_024923 | No sequencing reads – not detected in sample | | | | |
| OLIG3 | NM_175747 | No sequencing reads – not detected in sample | | | | |
| OVOL1 | NM_004561 | No sequencing reads – not detected in sample | | | | |
| PANX3 | NM_052959 | No sequencing reads – not detected in sample | | | | |
| PAX5 | NM_016734 | No sequencing reads – not detected in sample | | | | |
| PDZD2 | NM_178140 | No sequencing reads – not detected in sample | | | | |
| PECAM1 | NM_000442 | No sequencing reads – not detected in sample | | | | |
| PLAC4 | NM_182832 | No sequencing reads – not detected in sample | | | | |
| POU2AF1 | NM_006235 | No sequencing reads – not detected in sample | | | | |
| PROK1 | NM_032414 | No sequencing reads – not detected in sample | | | | |
| RAB26 | NM_014353 | No sequencing reads – not detected in sample | | | | |
| RPE65 | NM_000329 | No sequencing reads – not detected in sample | | | | |
| RPL30 | NM_000989 | No sequencing reads – not detected in sample | | | | |
| RPL37A | NM_000998 | No sequencing reads – not detected in sample | | | | |
| SCARA5 | NM_173833 | No sequencing reads – not detected in sample | | | | |
| SCRT1 | NM_031309 | No sequencing reads – not detected in sample | | | | |
| SERTM1 | NM_203451 | No sequencing reads – not detected in sample | | | | |
| SLC24A2 | NM_020344 | No sequencing reads – not detected in sample | | | | |
| SLC6A1 | NM_003042 | No sequencing reads – not detected in sample | | | | |
| SLC6A20 | NM_020208 | No sequencing reads – not detected in sample | | | | |
| SLC8A3 | NM_183002 | No sequencing reads – not detected in sample | | | | |
| SLCO4C1 | NM_180991 | No sequencing reads – not detected in sample | | | | |
| SSX1 | NM_005635 | No sequencing reads – not detected in sample | | | | |
| SV2B | NM_014848 | No sequencing reads – not detected in sample | | | | |
| SYK | NM_003177 | No sequencing reads – not detected in sample | | | | |
| TLX1 | NM_005521 | No sequencing reads – not detected in sample | | | | |
| TMEM105 | NM_178520 | No sequencing reads – not detected in sample | | | | |
| TNFAIP8L1 | NM_001167942 | No sequencing reads – not detected in sample | | | | |
| TP73 | NM_005427 | No sequencing reads – not detected in sample | | | | |
| TREML2 | NM_024807 | No sequencing reads – not detected in sample | | | | |
| TTC24 | NM_001105669 | No sequencing reads – not detected in sample | | | | |
| TUSC5 | NM_172367 | No sequencing reads – not detected in sample | | | | |
| UBC | NM_021009 | No sequencing reads – not detected in sample | | | | |
| UBE2QL1 | NM_001145161 | No sequencing reads – not detected in sample | | | | |
| UNC5A | NM_133369 | No sequencing reads – not detected in sample | | | | |
| USH2A | NM_206933 | No sequencing reads – not detected in sample | | | | |
| WSCD1 | NM_015253 | No sequencing reads – not detected in sample | | | | |
| YWHAZ | NM_145690 | No sequencing reads – not detected in sample | | | | |

p-value and FDR adjusted p-value are results of miR-negative control vs miR-210 transfected EVT cells, NA – adjusted p-value could not be calculated based on low read counts (<200), red shading denotes genes with a significant adjusted p-value (p<0.05), grey shading denotes genes with no sequencing reads
